# Identification of Fungicide Combinations for Overcoming *Plasmopara viticola* and *Botrytis cinerea* Fungicide Resistance

**DOI:** 10.3390/microorganisms11122966

**Published:** 2023-12-12

**Authors:** Junrui Zhang, Jhulia Gelain, Guido Schnabel, Samavath Mallawarachchi, Haoqi Wang, Nirmitee Mulgaonkar, Raghupathy Karthikeyan, Sandun Fernando

**Affiliations:** 1 Department of Biological and Agricultural Engineering, Texas A&M University, College Station, TX 77845, USA; 2 Department of Plant and Environmental Sciences, Clemson University, Clemson, SC 29634, USA; 3 Department of Agricultural Sciences, Clemson University, Clemson, SC 29634, USA

**Keywords:** fungicides, fungicide resistance, fungicide combinations, *Plasmopara viticola*, *Botrytis cinerea*, cytochrome b, molecular dynamics

## Abstract

Fungal diseases, including downy mildew (caused by *Plasmopara viticola*) and gray mold (caused by *Botrytis cinerea*), significantly impact the marketable yield of grapes produced worldwide. Cytochrome b of the mitochondrial respiratory chain of these two fungi is a key target for Quinone outside inhibitor (QoI)-based fungicide development. Since the mode of action (MOA) of QoI fungicides is restricted to a single site, the extensive usage of these fungicides has resulted in fungicide resistance. The use of fungicide combinations with multiple targets is an effective way to counter and slow down the development of fungicide resistance. Due to the high cost of in planta trials, in silico techniques can be used for the rapid screening of potential fungicides. In this study, a combination of in silico simulations that include Schrödinger Glide docking, molecular dynamics, and Molecular Mechanism-Generalized Born Surface Area calculation were used to screen the most potent QoI and non-QoI-based fungicide combinations to wild-type, G143A-mutated, F129L-mutated, and double-mutated versions that had both G143A and F129L mutations of fungal cytochrome b. In silico docking studies indicated that mandestrobin, famoxadone, captan, and thiram have a high affinity toward WT cytochrome b of *Botrytis cinerea*. Although the QoIs mandestrobin and famoxadone were effective for WT based on in vitro results, they were not broadly effective against G143A-mutated isolates. Famoxadone was only effective against one isolate with G143A-mutated cytochrome b. The non-QoI fungicides thiram and captan were effective against both WT and isolates with G143A-mutated cytochrome b. Follow-up in silico docking and molecular dynamics studies suggested that fungicide combinations consisting of famoxadone, mandestrobin, fenamidone, and thiram should be considered in field testing targeting *Plasmopara viticola* and *Botrytis cinerea* fungicide resistance.

## 1. Introduction

Grapes play a significant role in the global economy. In 2020, approximately 78 million tons of grapes were produced in an area of 6.95 million hectares, with a total value of over USD 80 billion [1,2]. Fungal diseases are a significant threat that impact the quality and quantity of grapes and may cause yield losses of up to 40% [3]. Moreover, fungal strains have started developing resistance to commonly used fungicides, which is becoming a serious concern that requires immediate attention.

Downy mildew, caused by the pathogen *Plasmopara viticola*, can cause severe damage to grapevines. The disease originated in North America and was introduced to Europe in the late 1800s, causing significant damage to European vineyards [4]. When conditions (high humidity and moderate temperature) are favorable, the pathogen invades the plant cells and obtains nutrients to produce sporangia, establishing new infections in the vineyard [4,5]. Leaves, shoots, and young berries are primarily impacted. As the infection progresses, yellow spots accompanied by white downy mold occur on the surfaces of the leaves, and the spots turn brown, eventually leading to necrosis [6]. Since young shoots and berries are susceptible to *Plasmopara viticola*, the infection leads to the distortion of young shoots and disrupts the normal translocation of water and nutrients, slowing growth [6]. The berries may become dehydrated or deformed and eventually fall [6]. The pathogen releases spores under abundant rainfall and warm temperatures, infecting large swaths of areas [6,7]. While QoI fungicides are generally effective in managing downy mildew, the risk of developing resistance to these fungicides is high as a result of mutations to the cytochrome b target [6,8,9]. 

Gray mold, another common fungal disease that results in significant crop losses, is caused by *Botrytis cinerea*. *Molecular Plant Pathology* named gray mold as one of the “Top 10 fungal plant pathogenic diseases” because of its diverse host range and its ability to invade different parts of the plant at different growth stages [10]. The berries of grapevines are vulnerable when rainfall is abundant under a moderate temperature [11]. Reddish-brown and watery decay can be observed as a symptom from the pedicel to the stylar end on the infected berries [11]. Infected regions are vulnerable sites for secondary infections, and these regions will generate more sporangia, leading to new infections of other berries nearby [11]. When berries are infected, they prematurely dry out. Brown lesions can be observed when *Botrytis cinerea* invades the leaves, flowers, and shoots of grapevines [11]. QoI fungicides are used for the chemical control of *Botrytis cinerea* either alone or in mixture [11]. However, the emergence of resistance to common QoI fungicides has severely limited its usefulness and is an ongoing concern. 

Cytochrome b is a protein within the cytochrome bc_1_ complex in *Plasmopara viticola* and *Botrytis cinerea,* and it is the major target of QoI fungicides. QoIs work by binding to cytochrome b and restricting respiratory function [12] and fungal reproduction [13]. When QoI fungicides bind to cytochrome b, electron transfer within cytochrome b and cytochrome c_1_ is disrupted, thus restricting the activity of ubiquinol oxidase substrate and inhibiting the production of ATP [8,12]. Without enough ATP, the propagation of the pathogen is interrupted. However, since QoI fungicides specifically bind to only a single target, i.e., cytochrome b, the risk of developing resistance is high, especially after the extensive usage of QoI fungicides [8,14,15]. QoI fungicides have been identified as high-risk fungicides by the Fungicide Resistance Action Committee (FRAC). Two main known mutations in downy mildew or gray mold, G143A (Glycine to Alanine) and F129L (Phenylalanine to Leucine), weaken the binding affinity between protein and fungicides, reducing the efficacy of QoI fungicides [8,9,16]. While creating novel fungicides can be a potential solution to fungicide resistance, the process takes significant amounts of time and resources. Meanwhile, applying fungicide combinations may be a viable and cost-effective pest management practice. Generally, a fungicide combination involves using one or more high-risk fungicides and one or more low-risk fungicides from existing fungicides [9]. Thus, fungicide combinations involving QoI fungicides and low-risk fungicides that can target multiple binding sites have the potential to be effective against developing resistance [9].

One of the main challenges associated with developing new fungicides or identifying suitable fungicide combinations to combat the fungicide resistance of plant diseases is the high cost and time required for in planta trials [17,18]. In silico techniques can be used for the rapid screening of existing antimicrobial compounds or the development of new antimicrobial compounds, which can significantly reduce the financial and labor costs of the subsequent steps [19,20,21]. In silico techniques such as molecular docking and molecular dynamics have been successfully used to design and identify antimicrobial peptides against multiple plant pathogens, including bacteria and fungi [22,23,24].

Only a few studies have addressed the selection of fungicide combinations for QoIs based on molecular structures and the molecular-level affinity of the fungicides to the cytochrome b active site [25]. In one of our previous studies [25], we identified some fungicide combinations using a machine learning algorithm. However, none of the combinations were experimentally tested. In this study, we provide a thermodynamic-based quantitative strategy to identify and select antifungal agents from QoIs (high-risk group) to be combined with low-risk fungicides to form fungicide combinations that can potentially mitigate fungicide resistance. During this process, selected fungicides from QoIs and the low-risk non-QoI group were docked with a model of fungal cytochrome b to find the fungicides with the highest affinity, further screened using molecular dynamics simulations, as well as MM-GBSA energy calculations, and validated using experimental data.

## 2. Materials and Methods

### 2.1. Protein Structure and Ligand Structure Preparation

A homology model for *Plasmopara viticola* (GenBank: DQ209286.1) was generated using the SWISS-MODEL server using cytochrome b from plant mitochondrial complex III2 from *Viga radiata* (PDB: 7JRG. 1 .C) as a template [26,27,28,29]. The quality of the model was evaluated via ERRAT and PROVE on the SAVES v6.0 server (https://saves.mbi.ucla.edu accessed on 8 January 2023) [30,31]. The homology model contained G143 and F129, which are key residues of WT cytochrome b. This model was mutated into three other versions on Maestro Schrödinger: G143A, F129L, and a mutation containing both G143 and F129L mutations. The canonical SMILES formulas of the ligands were obtained from the ZINC15 or PubChem databases (structural data on all the fungicides used in this study are provided under Supplementary Data Table S1), and an online SMILES translator was utilized to generate the 3D structures of these ligands [32,33]. Protein Preparation Wizard was used to prepare all the protein and ligand structures. This process involved adding missing hydrogens, correcting bond orders, fixing missing segments, and minimization under the Optimized Potentials for Liquid Simulations 3 (OPLS3) force field [34]. 

A homology model was constructed and validated for *Botrytis cinerea* using procedures analogous to those used for *Plasmopara viticola.*

### 2.2. Molecular Docking

Schrödinger Glide was used for docking the ligands on cytochrome b. The grid box was centered around the original active sites (G143 and F129; coordinates X—195.53, Y—213.29, Z —176.3) or mutated active sites (G143A; coordinates X—192.6, Y—212.54, Z—171.55; F129L; coordinates X—196.55, Y—213.55, Z—177.96; or F129L with G143A; coordinates X—195.53, Y—213.29, Z—176.3), and the dimensions of the grid box were 44 × 46 × 56 Å. The Glide docking scores for cytochrome b and 27 ligands (ubiquinol and the 26 fungicides shown in Table 1) were evaluated using Schrödinger Glide XP mode with default settings in three replicates. Binding affinity analysis was conducted based on the highest binding scores. Interactions between the ligands and the protein were analyzed using a ligand interaction diagram.

The in silico methods described in Section 2.1 and Section 2.2 are similar to those used in our previous studies [25].

### 2.3. Molecular Dynamics Simulations

Molecular dynamics (MD) simulations were conducted, as needed, to select fungicides using Schrödinger Desmond for further verification. The protein–ligand structures were created by merging the protein with a selective ligand. An orthorhombic box (distance 10 × 10 × 10 Å) in a Transferable Intermolecular Potential with 3 Points (TIP3P) solvent model was generated using the System Builder of Schrödinger Desmond under an OPLS3 force field. The charge of this system was kept in a neutral state by adding NaCl at 0.15 M concentration [34,35]. The NPT (normal pressure and temperature) ensemble was applied for the MD simulations with temperature at 300 K and pressure at 1.01325 bar [36]. Each protein–ligand structure within the orthorhombic box contained around 33,161 atoms with 9798 water molecules (data from the structure of cytochrome b with ubiquinol). The MD simulation for each system was run for 500 nanoseconds (ns) and generated 1000 frames at an interval of 500 picoseconds (ps). Root Mean Square Deviation (RMSD), Root Mean Square Fluctuation (RMSF), and protein–ligand contacts for each simulation were analyzed using Schrödinger Simulation Interaction Diagrams.

### 2.4. Binding Free Energy Analysis

Molecular Mechanism-Generalized Born Surface Area (MMGBSA) calculation was used to determine the binding affinity between protein and ligands based on the binding free energy [37]. By using the *thermal_MMGBSA.py* script from Schrödinger Prime for MD simulation with 1000 frames, the binding free energy of each frame or each segment, depending on command *-step_size* on the script, was calculated to analyze binding affinity [37].

### 2.5. In Vitro Experiments 

#### 2.5.1. *Botrytis cinerea* Isolates

A total of 6 isolates of *B. cinerea* were included in the sensitivity of conidia germination assays (Table 2). These isolates were obtained from symptomatic strawberry fruit in 2011 and characterized previously as sensitive (S) or resistant (R) to the QoI fungicide pyraclostrobin by the authors of [38] through a discriminating dose assay (10 µg/mL). The G143A mutation in the *CYTB* gene was only found in the R isolates, detected using the restriction fragment length polymorphism (RFLP) method [38,39].

A total of 4 QoI fungicides (FRAC11) were tested, including azoxystrobin, famoxadone, fenamidone, and mandestrobin. The Quinone outside Inhibitor stigmatellin binding-type (QoSI) fungicide ametoctradin (FRAC 45) and the multisites thiram (FRAC M03) and captan (FRAC M04) were also tested. The list of active ingredients, FRAC codes, commercial names, companies, and fungicide concentrations used in the germination inhibition assays is listed in Table 3. Except for famoxadone, which was used in its original concentration, stock solutions were prepared by dissolving the fungicides in water. A stock solution of salicylhydroxamic acid (SHAM, 99% a.i.; Sigma-Aldrich Inc., St. Louis, MO, USA) was prepared at a concentration of 50,000 µg/mL in methanol.

Sensitivity of conidial germination. Abundant sporulation was obtained from canned peaches in peach juice placed in sterile plastic pots, inoculated with mycelial plugs containing *B. cinerea* mycelium, and incubated at 25 °C with a 12 h photoperiod for 7 days. To make conidial suspensions, spores were collected with the aid of autoclaved toothpicks and placed in Eppendorf tubes containing 2 mL of autoclaved tap water. The conidia suspensions were adjusted to 5 × 10^5^ conidia/mL using a hemacytometer. Stock solutions of fungicides were prepared as described above and added into 2% water agar (WA) to produce the concentrations listed in Table 3. SHAM at 100 µg/mL was added into the QoI and QoSI-amended plates and their respective control plates. A conidial suspension (50 μL) of each isolate was spread with the aid of L-shaped spreaders on a Petri plate containing 2% WA amended with each fungicide. Plates were incubated at 20 °C for 12 h. One plate was used for each treatment. Germination percentage was then determined by counting 100 conidia on each half of the plate (n = 200) under a microscope, scored as either non-germinated or germinated, and EC_50_ values were calculated. A conidium was considered germinated if the germ tube was at least twice the length of the conidium. This experiment was conducted twice.

#### 2.5.2. Sensitivity of Mycelial Growth for Ametoctradin

A mycelial growth inhibition assay was used to determine the EC_50_ values of ametoctradin for the 6 *B. cinerea* isolates. We prepared V8 juice agar plates (100 mL V8 juice, 1.4 g calcium carbonate (CaCO_3_), and 15 g agar) that were 90 mm in diameter and amended them with 0.04, 0.08, 0.2, 0.4, 0.8, or 1 μg mL^−1^ ametoctradin. SHAM was added to the medium at a concentration of 100 μg/mL to inhibit the alternative respiration pathway. Fresh plugs (5 mm in diameter) were cut from the growing edge of active mycelia and placed on the fungicide-amended media. The diameter of each colony was measured perpendicularly after 3 days of incubation at 20 °C in the dark. Two plates containing two plugs each were used as replicates, and the experiment was repeated twice.

## 3. Results and Discussion

### 3.1. Fungicide Binding Behavior on Botrytis cinerea Cytochrome b

To identify the binding affinities between the selected QoI fungicides and cytochrome b of *Botrytis cinerea*, docking simulations were performed using a grid box covering the ubiquinol binding site and the specific residues G143 and F129 on cytochrome b. *Botrytis cinerea* was selected for this initial study primarily because *Plasmopara viticola* is an obligate parasite, and experimental validations could only be performed under field conditions, whereas *Botrytis cinerea* validations could easily be carried out in a laboratory setting.

#### 3.1.1. General Observations

**Figure 1:**
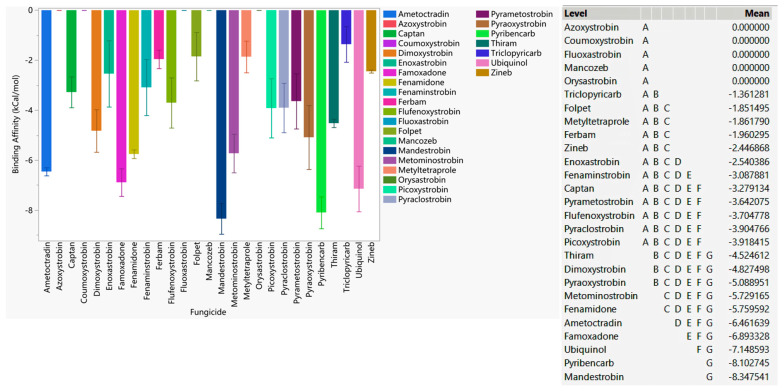
The average performances of select QoI fungicides on WT, G143A-mutated, F129L-mutated, and G143A–F129L double-mutated cytochrome b of *Botrytis cinerea*. Mean—average binding affinity of three replicates (generated by Glide docking on Maestro Schrödinger) of the corresponding ligand; level—ligands with the same letter are not significantly different (letter level A: ligands with the lowest binding affinity; letter level G: ligands with the highest binding affinity; the binding affinity ranges from G to A).

Our comparison of binding affinities against WT, F129L, G143A, and F129L-G143A double-mutated cytochrome b of *Botrytis cinerea* revealed that mandestrobin and pyribencarb had a higher binding affinity than ubiquinol, indicating that these fungicides have a better likelihood of being effective against inhibiting cytochrome b of *Botrytis cinerea* in general (Figure 1). Famoxadone, fenamidone, ametoctradin, metominostrobin, pyraoxystrobin, dimoxystrobin, and thiram had higher affinities than the others, indicating their broad-spectrum ability to bind to the active site regardless of the occurrence of the two common mutations. It should also be noted that azoxystrobin, identified as a resistant fungicide, neither bound to WT nor to any of the mutated versions. Since coumuxystrobin, fluoxastrobin, and orysastrobin did not bind to WT or any of the mutated versions, these fungicides are considered to be at the highest risk of developing resistance. Fungicides such as folpet, ferbam, zineb, and captan, which were categorized as low-risk, did not bind with appreciable affinity to the cytochrome b active site, indicating that they may not be active via QoI mode of action (MOA).

#### 3.1.2. Mutation-Specific Observations

##### Fungicide Recommendations for G143A Mutation

**Figure 2:**
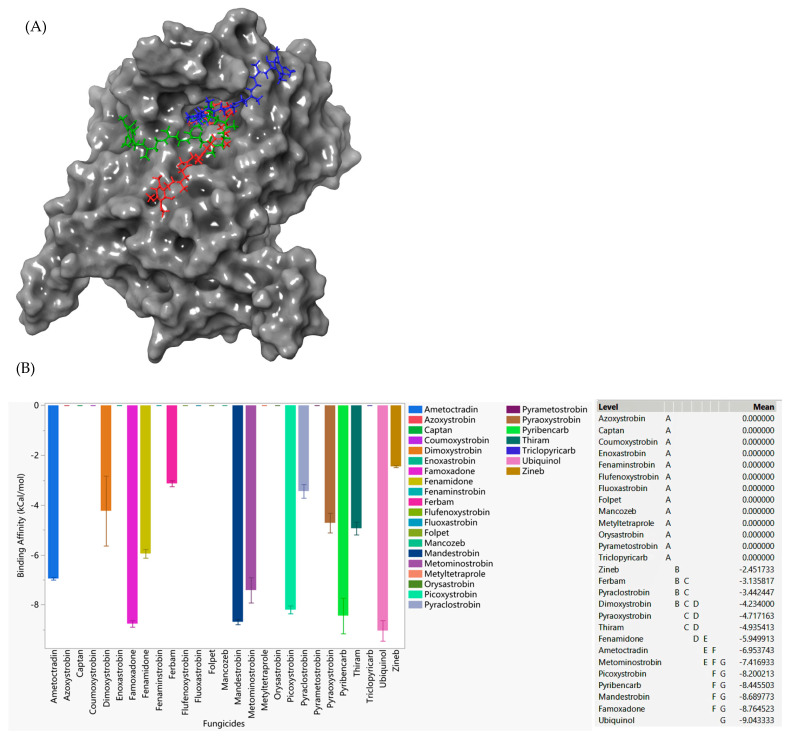
(**A**) Three potential binding poses of ubiquinol with the G143A-mutated cytochrome b of *Botrytis cinerea* (red/green/blue sticks—ubiquinol; G143A-mutated cytochrome b—gray surface). (**B**) The performances of select QoI fungicides on the G143A-mutated cytochrome b of *Botrytis cinerea* in a specific grid box. Mean—average binding affinity of three replicates (generated by Glide docking on Maestro Schrödinger) of the corresponding ligand; level—ligands with the same letter are not significantly different (letter level A: ligands with the lowest binding affinity indicating the ligand does not bind to the particular site; letter level G: ligands with the highest binding affinity; the binding affinity ranges from G to A).

Three potential binding conformations of ubiquinol with G143A-mutated cytochrome b are shown in Figure 2A. The binding affinities of the red, green, and blue sticks were −9.861 kCal/mol, −8.741 kcal/mol, and −8.528 kCal/mol, respectively. Our docking studies revealed that ubiquinol had the strongest affinity to the G143A-mutated cytochrome b of *Botrytis cinerea* (Figure 2B). Virtually all the QoI fungicides considered in this study had a lower affinity to cytochrome b than the ubiquinol control, indicating their weak ability to succeed against competitive inhibition. Only famoxadone had a close enough affinity to the site in comparison with ubiquinol, indicating its potential to withstand resistance (Figure 2). Coumoxystrobin, fluoxastrobin, metyltertrapole, and orysastrobin did not bind to the G143A cytochrome b. Azoxystrobin, identified as a resistant fungicide, did not bind to the G143A cytochrome b. Ferbam and zineb had weaker binding affinity, while captan and folpet did not bind to the G143A cytochrome b, indicating that these four low-risk fungicides were likely not effective against the G143A-mutated cytochrome b of *Botrytis cinerea*. Among the low-risk fungicides, Thiram showed the strongest affinity. 

**Figure 3:**
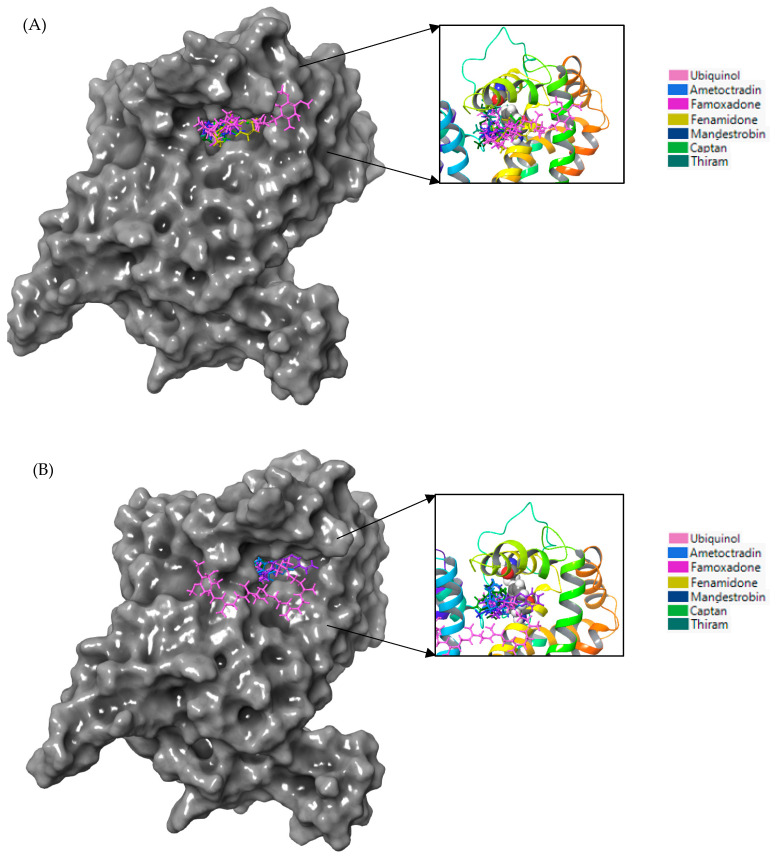
(**A**) Ametoctradin, mandestrobin, famoxadone, fenamidone, thiram, captan, and ubiquinol are visualized as sticks with different colors and bound to WT cytochrome b (gray surface). (**B**) Ametoctradin, mandestrobin, famoxadone, fenamidone, thiram, captan, and ubiquinol (sticks) bound to the G143A cytochrome b (gray surface) of *Botrytis cinerea*. The figure to the right represents a close-up of the active site, with the protein represented as a rainbow-colored ribbon and F129 and G143 or G143A represented as ball structures.

An analysis of the binding behavior of ametoctradin, mandestrobin, famoxadone, fenamidone, thiram, and captan at the vicinity of the G143 and F129 residues of WT cytochrome b of *Botrytis cinerea* indicated that they all bound close to the two residues (Figure 3A). Despite having a lower affinity compared to the native substrate ubiquinol, for the G143A mutation, ametoctradin, mandestrobin, famoxadone, fenamidone, thiram, and captan bound to the same site as WT cytochrome b, indicating that this site was crucial when selecting effective QoIs for the targeting of *Botrytis cinerea* cytochrome b inhibition. 

**Figure 4:**
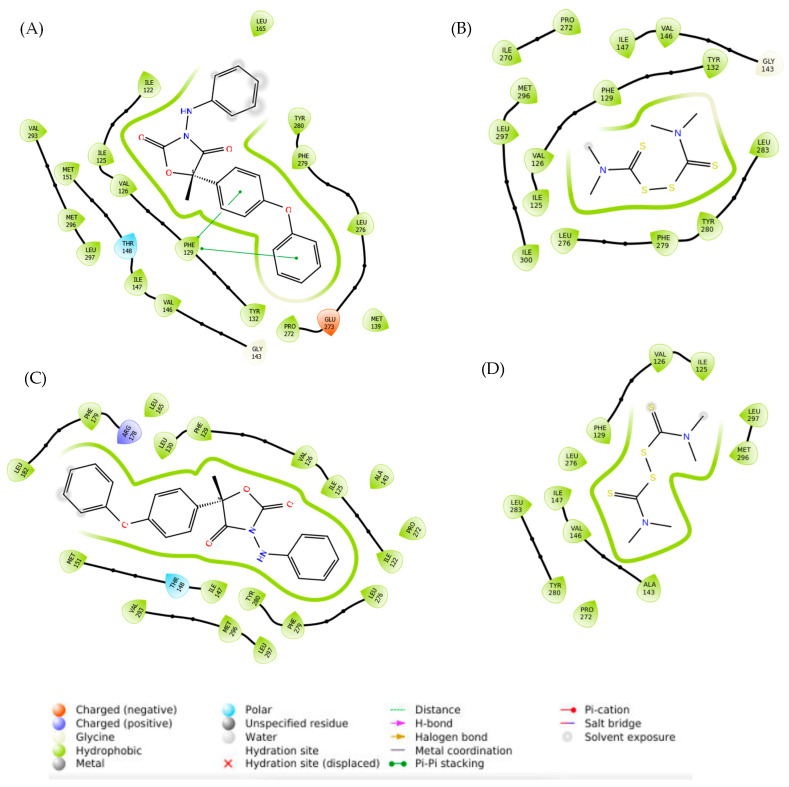
Interactions of (**A**) famoxadone and (**B**) thiram with WT cytochrome b. (**C**) Interactions of famoxadone and (**D**) thiram with the G143A-mutated cytochrome b of *Botrytis cinerea*.

Our analysis of the interactions (Figure 4) of cytochrome b of WT *Botrytis cinerea* with famoxadone and thiram indicated that hydrophobic bonding was the primary interaction that occurred. For the G143A-mutated cytochrome b, hydrophobic bonding still played a major role in the interaction of famoxadone and thiram. The interaction with the residue F129 was hydrophobic regardless of the ligand. The interactions of ligands with the residue G143 were not apparent in WT cytochrome b. However, famoxadone and thiram showed hydrophobic bonding once the G143A mutation occurred on *Botrytis cinerea* cytochrome b.

### 3.2. In Vitro Experimental Results

Sensitivity of conidial germination. The EC_50_ values obtained based on conidia germination inhibition are presented in Table 4. For the QoI fungicides (FRAC 11) azoxystrobin, famoxadone, and mandestrobin, all three isolates without the G143A mutation presented EC_50_ values < 0.3 µg/mL, while the three isolates with the G143A mutation presented EC_50_ values > 100 µg/mL, except for isolate NC7 for famoxadone, which presented an EC_50_ value of 2.908 µg/mL for famoxadone. All isolates, regardless of whether they had the G143A point mutation, had estimated EC_50_ values > 100 µg/mL for the QoSI fungicide ametoctradin in the germination inhibition assay. The isolates of *Botrytis cinerea* tested had estimated EC_50_ values < 1 µg/mL for the multisites captan and thiram. 

The effective concentration that inhibits fungal growth by 50% relative to the control (EC_50_) values, in µg/mL, of azoxystrobin, famoxadone, fenamidone, mandestrobin, ametoctradin, captan, and thiram for six isolates of *Botrytis cinerea* are shown in Table 4.

It was observed that, for WT, azoxystrobin, famoxadone, and mandestrobin QoIs, as well as thiram (a LR non-QoI), showed a high affinity toward cytochrome b via in silico simulations, and these results agree with experimental observations. Ametoctradin was identified as a low-affinity fungicide across the board, which was confirmed by in vitro studies. In silico studies accurately predicted fenamidone as a non-effective fungicide for WT in general since the affinity (−5.5 kCal/mol) was lower (i.e., higher free energy) than that for ubiquinol (−6.0 kCal/mol). 

For isolates with the G143A mutation, the non-QoI LR fungicide thiram was the only fungicide that agreed with simulation predictions, indicating that a single-target approach may not be effective against the G143A mutation and that a multiple-target approach is likely the most promising. 

Experimental validations revealed captan to be effective for the G143A-mutated cytochrome b, but this was not predicted in the simulations. Most likely, the reason for this is that the targets for captan are different from cytochrome b, and captan inhibits the fungus via a different MOA.

### 3.3. Fungicide Binding Behavior on Plasmopara viticola Cytochrome b

#### 3.3.1. General Observations

Since the focus of this study was to identify fungicides that were effective against multiple mutations of the cytochrome b, the same set of QoI fungicides were docked onto the four variations of *Plasmopara viticola* cytochrome b, namely the WT, G143A-mutated, F129L-mutated, and double-mutated variations of *Plasmopara viticola* cytochrome b, which included both the G143A and F129L mutations. Here, G143A and F129L double mutations were specifically selected, since those mutations were reported to be most significant for antifungal resistance [27]. 

**Figure 5:**
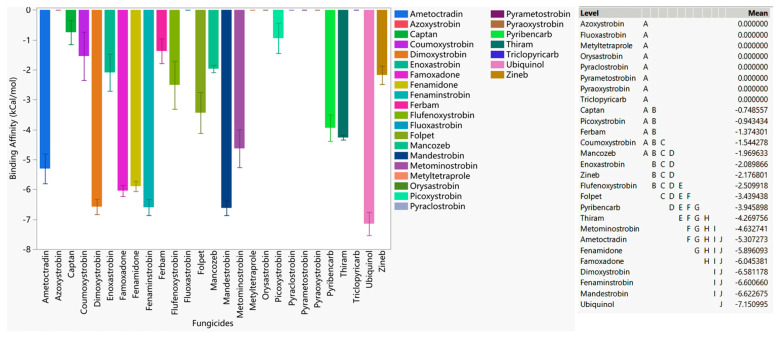
The performances of select QoI fungicides on the WT, G143A-mutated, F129L-mutated, and double-mutated cytochrome b of *Plasmopara viticola* in general. Mean—average binding affinity of three replicates (generated by Glide docking on Maestro Schrödinger) of the corresponding ligand; level—ligands with the same letter are not significantly different (letter level A: ligands with the lowest binding affinity; letter level J: ligands with the highest binding affinity; the binding affinity ranges from J to A).

In order to capture the effectiveness of fungicides on different forms of *Plasmopara viticola* cytochrome b, a general statistical analysis was performed (Figure 5). Here, ubiquinol, as a native substrate, had the highest binding affinity toward the WT, G143A-mutated, and F129L-mutated types of cytochrome b of *Plasmopara viticola*. From high-risk QoI fungicides, mandestrobin, fenaminstrobin, dimoxystrobin, famoxadone, fenamidone, and ametoctradin emerged as those with the strongest affinity toward *Plasmopara viticola* cytochrome b; however, all these QoIs have a lower affinity to all forms of cytochrome b, indicating their relative weakness as a competitive inhibitor against ubiquinol and their high propensity to be susceptible to developing resistance. 

Pyraoxystrobin, pyrametostrobin, pyraclostrobin, triclopyricarb, orysastrobin, fluoxastrobin, and metyltetraprole did not bind (i.e., they had the lowest affinity) to all forms of cytochrome b, indicating high susceptibility to possible resistance. Azoxystrobin, which has already been identified as a resistant fungicide to *Plasmopara viticola* cytochrome b in certain regions, did not bind to any version of cytochrome b, corroborating field observations [40]. The non-QoI thiram showed a stronger binding affinity than the other low-risk fungicides, indicating its potentially superior efficacy against cytochrome b among the those in the low-risk category. Fungicides folpet, zineb, mancozeb, ferbam, and captan showed weaker affinity or did not bind to the protein, meaning these low-risk fungicides had a different MOA to cytochrome b inhibition. 

A common recommendation is to use fungicide combinations that consist of different modes of actions; i.e., combining one MOA with others in a fungicide rotation program. Due to their ability to tackle multiple mutations based on in silico docking simulations, fenamidone, famoxadone, mandestrobin, dimoxystrobin, fenaminstrobin, ametoctradin QoIs, and the non-QoI, thiram are suitable candidates that should be considered for field testing in rotational programs targeting *Plasmopara viticola*. 

#### 3.3.2. Mutation-Specific Observations

##### Fungicide Recommendations for the G143A-F129L Double Mutation

**Figure 6:**
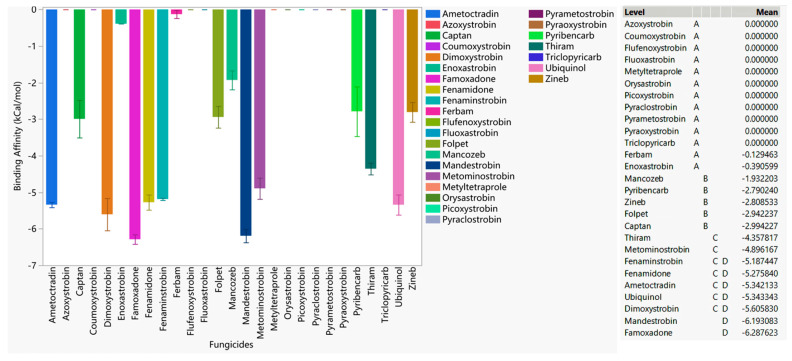
The performances of select QoI fungicides on the F129L-G143A double-mutated cytochrome b of *Plasmopara viticola* in a specific grid box. Mean—average binding affinity of three replicates (generated by Glide docking on Maestro Schrödinger) of the corresponding ligand; level—ligands with the same letter are not significantly different (letter level A: ligands with the lowest binding affinity; letter level D: ligands with the highest binding affinity; the binding affinity ranges from D to A).

Unlike the interaction with WT, F129L, and G143A-mutated cytochrome b, ubiquinol did not show the highest binding affinity toward cytochrome b when the F129L-G143A double mutation occurred (Figure 6). Famoxadone, mandestrobin, and dimoxystrobin showed a higher affinity to double-mutated cytochrome b, indicating their potential superiority against double-mutated cytochrome b. Ametoctradin, fenamidone, fenaminstrobin, and metominostrobin also had a higher affinity to the double-mutated site compared to the other fungicides. Coumoxystrobin, picoxystrobin, and flufenoxystrobin did not bind to the double-mutated cytochrome b. Pyraoxystrobin, pyrametostrobin, pyraclostrobin, triclopyricarb, orysastrobin, fluoxastrobin, and metyltetraprole did not bind to any type of cytochrome b, indicating their high propensity to be resistant. Moreover, binding affinity calculations on cytochrome b of WT, G143A-mutated, F129L-mutated, and G143A-F129L-mutated versions verified the tendency of azoxystrobin to be resistant. Only thiram showed higher affinity for the G143A-F129L double-mutated cytochrome b compared to the other low-risk fungicides analyzed (folpet, ferbam, zineb, mancozeb, and captan). 

**Figure 7:**
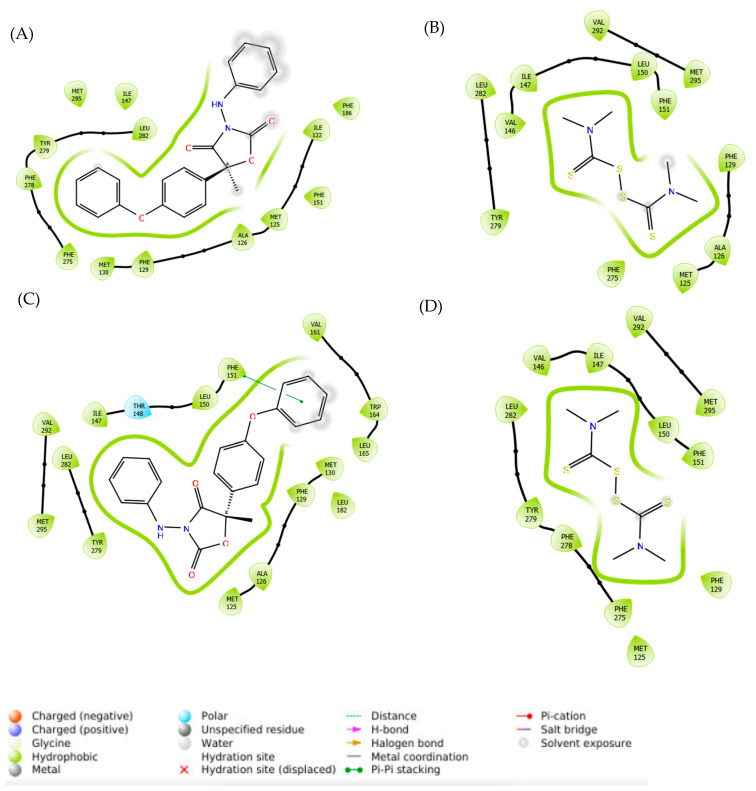
Binding interactions of (**A**) famoxadone and (**B**) thiram with WT cytochrome b; (**C**) binding interactions of famoxadone and (**D**) thiram with the G143A-mutated type of cytochrome b.

The binding conformations of famoxadone and thiram with the WT and G143A-mutated versions of cytochrome b are given in Figure 7. Famoxadone and thiram formed strong hydrophobic interactions with the WT and G143A-mutated versions of cytochrome b, indicating that the primary interactions between the fungicides and cytochrome b were hydrophobic, which agrees with the predominantly hydrophobic nature of the cytochrome b proteins [41,42]. Figure 7 shows two fungicides forming strong hydrophobic interactions with 122–151, 279–282, and 292–295 regions in the WT and G143A-mutated versions. 

**Figure 8:**
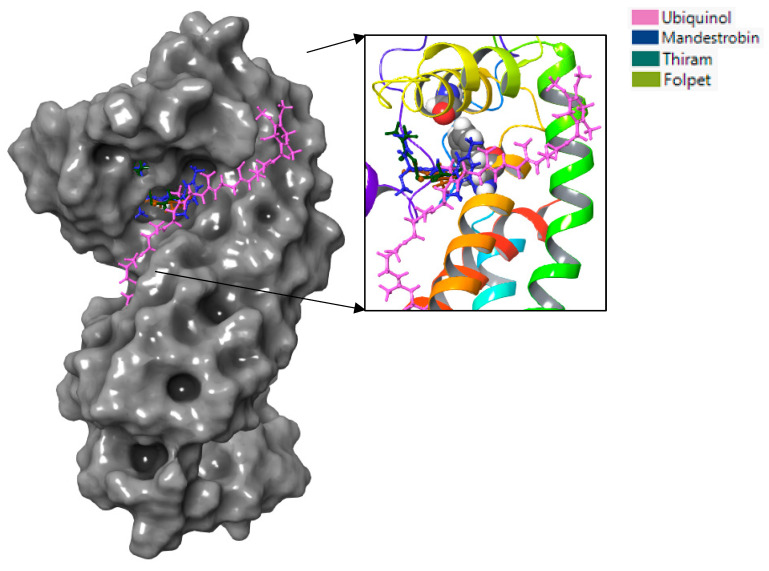
Mandestrobin, thiram, folpet, and ubiquinol are depicted as sticks with different colors and bound to the gray surface representing the WT cytochrome b of *Plasmopara viticola*. The figure to the right represents a close-up of the active site, with the protein depicted in a rainbow-colored ribbon and F129 and G143 shown as ball structures.

Based on the binding interaction analysis (Figure 8), the pocket located on the top cytochrome b region containing residues F129 and G143 seemed critical when targeting *Plasmopara viticola* inhibition. Mandestrobin, thiram, and folpet tended to bind to this pocket, overlapping with the native substrate ubiquinol. 

### 3.4. Molecular Dynamics Simulations with Plasmopara viticola Cytochrome b

To further evaluate the binding behavior of these fungicides to cytochrome b, MD simulations were carried out for selected antifungal agents on multiple mutated versions of cytochrome b. The Emodel and MM-GBSA energies for the selected antifungal agents (based on MD simulations) are given in Table 5. The energy values were calculated for various combinations of mutations.

According to the average binding free energy and Emodel values, ubiquinol, the native substrate, showed a strong affinity toward all versions of cytochrome b. When the simulation starting site was randomly confined to residues L123 and G137 or their mutated version(s) (L123F and G137A), the high-risk fungicides, including fenamidone, famoxadone, and mandestrobin, showed more negative binding free energy and Emodel values compared to the low-risk fungicides (thiram and captan), indicating that the QoI fungicides had stronger affinities compared to the low-risk, non-QoI ones. However, it was clear that all the fungicides tested had lower affinities than the native substrate ubiquinol, indicating the broader weakness of the tested fungicides against competitive inhibition. A similar behavior was observed when the active site was confined to F129 and G143 or their mutated version(s), with QoI fungicides showing slightly stronger binding affinities with cytochrome b but still significantly lower affinities compared to ubiquinol. Based on the binding free energies and Emodel values, fenamidone and famoxadone were identified as those with the strongest affinities to all mutated versions of cytochrome b, while mandestrobin and thiram showed better affinities only to some of the variations. 

**Figure 9:**
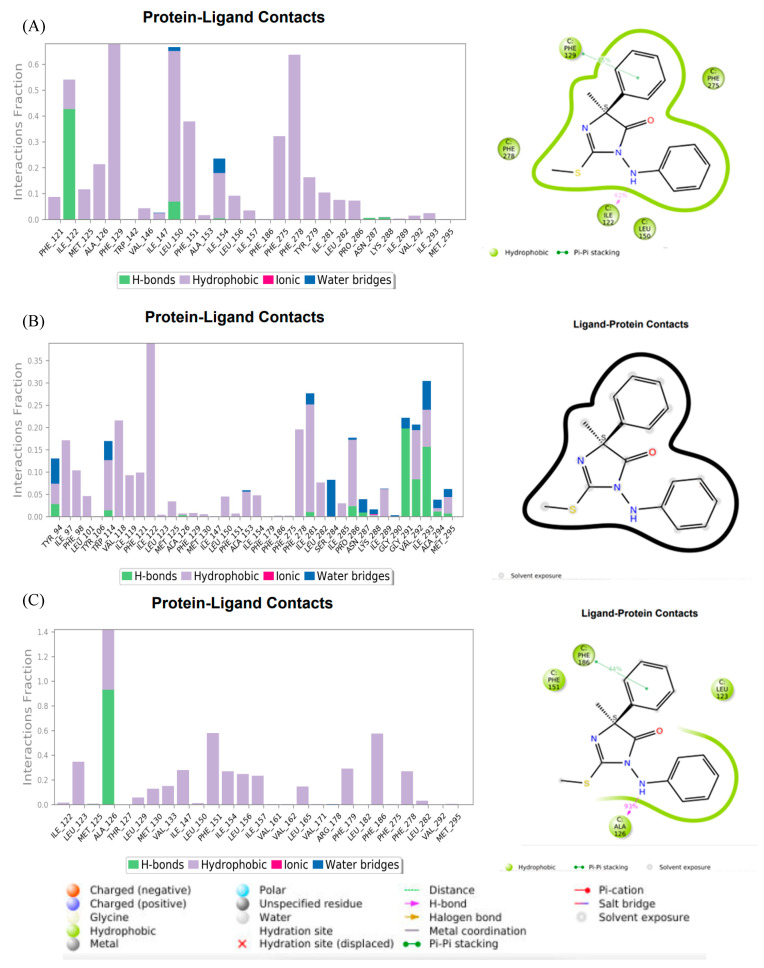
The interactions of fenamidone with the (**A**) WT, (**B**) G143A-mutated, and (**C**) F129L-mutated versions at the F129 and G143 binding sites of *Plasmopara viticola*. In the protein–ligand interaction sections, the y-axis shows the residues on the protein; the x-axis represents the simulation time regarding how long the ligands stayed on each residue. The inset to the right shows the ligand–protein interactions.

In order to obtain an in-depth understanding of how each ligand is bound to the target domain, the dominant interactions of ligands with cytochrome b during the MD simulations were analyzed. The interactions of fenamidone with the WT and F129L-mutated cytochrome b are given in Figure 9, and the interaction diagrams for other ligands are included in the Supplementary Data. Similar to the docking results, hydrophobic interactions were dominant between *Plasmopara viticola* cytochrome b and the ligands. The native substrate ubiquinol formed strong hydrophobic interactions with both the WT and F129L-mutated versions; however, significant changes in the interactions were observed for the G143A-mutated and double-mutated versions (Supplementary Figures S9–S12). For example, for the G143A-mutated version, ubiquinol formed strong hydrophobic interactions at PHE141 and hydrogen bonding at ALA260, while for the double-mutated versions, TYR94 and TRP273 were the main points of the hydrophobic interactions.

Other ligands also showed significant changes in their interactions as a result of the mutations. Fenamidone showed high robustness against mutations, forming strong hydrophobic interactions and hydrogen bonding in the regions of ILE122-PHE129 and PHE151-TRP164 in all mutated versions of cytochrome b. Famoxadone also showed strong interactions at these regions for all variations except for the G143A mutation, while it formed strong hydrogen bonding at ILE269 and TRY279 with the G143A-mutated version. This observation agrees with the relatively low (more negative) binding energies shown by fenamidone and famoxadone against all variations of cytochrome b. Mandestrobin formed strong hydrophobic interactions in the vicinity of TYR279 for all versions except the double-mutated version, while for the double-mutated version, the ILE119 was the major site forming hydrophobic interactions. This agrees with the binding energies of mandestrobin and suggests that mandestrobin has lower affinity towards the double-mutated version of cytochrome b compared to the other three versions. 

Among the low-risk fungicides, thiram showed strong hydrophobic interactions at the vicinity of PHE129 for both the WT and G143A-mutated versions and at PHE121 and PHE278 for the F129L- and double-mutated versions. However, the interactions of thiram were much weaker than the high-risk fungicides, which agrees with the less negative binding energies. While ametoctradin had strong hydrophobic binding at ILE147 and PHE151 with the WT protein, it had only weak hydrophobic interactions against the mutated versions, suggesting that it may not be effective against the mutations, which also agrees with the study by the authors of [43]. 

When the docking site was centered on L123 and G137, a randomly selected location, the interactions of the native substrate ubiquinol with the mutated versions changed significantly. However, both fenamidone and famoxadone showed strong hydrophobic interactions or hydrogen bonding at the ILE121-TYR132 regions of all variations, suggesting that these two compounds are also robust against these mutations. Similar to the F129- and G143-centered simulations, the interactions of famoxadone changed significantly under the G137A- and double-mutated versions. Thiram, captan, and azoxystrobin bound weakly against most of the mutated versions. Thus, based on the interactions during the MD simulations, fenamidone and famoxadone were identified as the most robust fungicides against all tested mutations. 

The stability of the binding interactions during the MD simulations was observed using RMSD diagrams. The simulations of ubiquinol, fenamidone, famoxadone, mandestrobin, and ametoctradin were equilibrated against all versions of cytochrome b, indicating stable binding with all mutated versions. However, thiram showed significant fluctuations against the F129L- and F129L-G143A mutated versions, suggesting its potential susceptibility to low affinity to these mutations on cytochrome b. Azoxystrobin also did not show stable binding with multiple mutations, which was expected, since it was resistant and ineffective against some mutations of cytochrome b. RMSD diagrams for the ligands against different cytochrome b variations are given in Supplementary data. Overall, the MD simulations further reinforced the findings from the docking analyses.

It should be noted that although some simulation results pertinent to *Botrytis cinerea* were verified via in vitro experiments, this was not the case for *Plasmapora viticola*. Accordingly, it is necessary to validate the recommended fungicides in planta. Also, the simulations did not consider fungicide concentrations; thus, further studies are needed to identify the best QoI and non-QoI combinations and their respective concentrations. 

## 4. Conclusions

The primary purpose of this study was to use in silico simulations to select the highest-affinity QoI and non-QoI fungicides to cytochrome b targets of *Plasmopara viticola* and *Botrytis cinerea*. Our in silico simulations showed that ubiquinol was the highest-affinity ligand for a majority of the variations of cytochrome b, regardless of the sourced organism. As a result, most of the QoIs and non-QoIs tested bound weakly to cytochrome b. In silico simulations with the WT, F129L-mutated, G143A-mutated, and G143A-F129L double-mutated cytochrome b of *Botrytis cinerea* revealed that mandestrobin and pyribencarb had a higher binding affinity than ubiquinol, indicating that these fungicides have a better likelihood of being effective against inhibiting cytochrome b of *Botrytis cinerea* in general. The non-QoI fungicide thiram had higher affinities than the other non-QoIs tested, indicating it broad-spectrum ability to bind to the active sites regardless of the occurrence of the two common mutations: G143A and F129L. Azoxystrobin, which has been identified as a resistance-prone fungicide, did not bind to WT or any of the mutated versions of *Botrytis cinerea* cytochrome b. Considering the G143A, F129L and G143A-F129L double mutations, among all the QoIs tested, only famoxadone had a close enough affinity compared with ubiquinol to the cytochrome b site, indicating its potential to withstand resistance. In vitro experiments revealed that the QoIs mandestrobin, famoxadone, and azoxystrobin and the non-QoIs captan and thiram were effective against FLOR5 (WT), HP9 (WT), and NC4 (WT) isolates of *Botrytis cinerea*. Famoxadone was the only QoI that was effective against the NC7 isolate, which was confirmed to have developed resistance to the QoI ametoctradin via the G143A mutation. However, famoxadone was not effective against two other ametoctradin-resistant isolates: FLOR8 and MOD12. The QoIs fenamidone and mandestrobin were not effective against any of the ametoctradin-resistant isolates. The non-QoI LR fungicides captan and thiram were effective for all forms of ametoctradin-resistant isolates with the G143A mutation. 

Simulations with *Plasmopara viticola* indicated that the native substrate ubiquinol bound to the WT, G143A-mutated, F129L-mutated, and G143A-F129L double-mutated cytochrome b with the highest affinity. Among the high-risk QoI fungicides, mandestrobin, fenaminstrobin, dimoxystrobin, famoxadone, fenamidone, and ametoctradin emerged as those with the strongest affinity toward *Plasmopara viticola* cytochrome b; however, all these QoIs had a lower affinity than ubiquinol to all forms of cytochrome b, indicating their relative weakness as a competitive inhibitor against ubiquinol and their high likelihood to be susceptible to developing resistance. Azoxystrobin, a QoI that has already been identified as resistant to *Plasmopara viticola* cytochrome b, did not bind to any version of cytochrome b, corroborating field observations. The non-QoI thiram showed a stronger binding affinity than the other low-risk fungicides to all forms of *Plasmopara viticola* cytochrome b, indicating its potentially superior efficacy among those in the low-risk category. Due to their ability to tackle multiple mutations based on in silico docking simulations, the QoIs fenamidone, famoxadone, mandestrobin, dimoxystrobin, fenaminstrobin, ametoctradin and the non-QoI thiram are suitable candidates that should be considered for field testing in rotational programs targeting *Plasmopara viticola*. Based on MD simulations, ubiquinol, the native substrate, showed a strong affinity toward all versions of *Plasmopara viticola* cytochrome b. Our MD simulations suggested that the QoIs fenamidone, famoxadone, and mandestrobin and the non-QoIs thiram and captan have the strongest affinities to the WT and mutated versions of *Plasmopara viticola* cytochrome b, suggesting the value of field testing combinations of these fungicides for the management of *Plasmopara viticola* fungicide resistance.

Overall, based on in silico and in vitro studies, fungicide combinations consisting of QoI fungicides such as famoxadone and mandestrobin and non-QoI fungicides like thiram and captan are the most promising fungicide combinations and are recommended for field trials.

## Figures and Tables

**Table 1:** Information on the resistance and modes of action of the fungicides selected for this study.

Fungicide	Resistance ^1^	Fungicide Type ^2^
Ubiquinol	NA	NA
Famoxadone	HR	QoI
Azoxystrobin	HR/R	QoI
Fenamidone	HR	QoI
Coumoxystrobin	HR	QoI
Flufenoxystrobin	HR	QoI
Enoxastrobin	HR	QoI
Pyraoxystrobin	HR	QoI
Picoxystrobin	HR	QoI
Metyltetraprole	HR	QoI
Fenaminstrobin	HR	QoI
Pyribencarb	HR	QoI
Dimoxystrobin	HR	QoI
Triclopyricarb	HR	QoI
Metominostrobin	HR	QoI
Pyrametostrobin	HR	QoI
Mandestrobin	HR	QoI
Fluoxastrobin	HR	QoI
Pyraclostrobin	HR	QoI
Orysastrobin	HR	QoI
Folpet	LR	PHT
Ferbam	LR	DTC
Captan	LR	PHT
Mancozeb	LR	DTC
Ametoctradin	HR/R	QoI
Thiram	LR	DTC
Zineb	LR	DTC

^1^ Resistance: NA—native, HR—high risk for the resistance of fungicides, LR—low risk for the resistance of fungicides. ^2^ Fungicide type: QoI—quinone outside inhibitor, DTC—dithiocarbamate, PHT—phthalimides.

**Table 2:** Codes, origin, presence, or absence of G143A mutation in the CYTB gene and the phenotypical sensitivity to pyraclostrobin of the *Botrytis cinerea* isolates used in this study.

Isolate Name	State	G143A Mutation	Pyraclostrobin Sensitivity
FLOR5	South Carolina	Absent	S
HP9	North Carolina	Absent	S
NC4	North Carolina	Absent	S
FLOR8	South Carolina	Present	R
MOD12	South Carolina	Present	R
NC7	North Carolina	Present	R

**Table 3:** Active ingredients used in the germination inhibition assays and their FRAC codes, commercial names, companies, and fungicide concentrations used in the germination inhibition assays.

Active Ingredient	FRAC Code	Commercial Name	Company	Fungicide Concentrations (µg/mL)							
Fenamidone	11	Reason 500SC 44.4%	Bayer CropScience	0	0.005	0.01	0.5	1	5	10	100
Azoxystrobin	11	Abound 22.9%	Syngenta	0	0.1	0.5	1	5	10	100	
Famoxadone	11	Famoxadone solution 100 μg/mL	Supelco	0	0.01	0.05	0.1	1			
Mandestrobin	11	Intuity 43.4%	Valent	0	0.01	0.1	0.5	10			
Ametoctradin	45	Zampro 20.20%	BASF	0	0.01	0.05	0.1	1	10		
Thiram	M03	Thiram SC 44%	Taminco/Sunland	0	0.05	0.1	1	10			
Captan	M04	Captan 80WD 80%	Drexel Chemical Company	0	0.05	0.1	1	10			

**Table 4:** Fungicide sensitivity of *Botrytis cinerea* isolates from North and South Carolina.

Isolate	EC_50_ values (µg/mL)						
	Azoxystrobin	Famoxadone	Fenamidone	Mandestrobin	Ametoctradin	Captan	Thiram
FLOR5	0.147	0.168	>100	0.026	>100	0.384	0.357
HP9	0.002	0.034	>100	0.011	>100	0.570	0.253
NC4	0.352	0.178	>100	0.003	>100	0.963	0.621
FLOR8	>100	>100	>100	>100	>100	0.709	0.566
MOD12	>100	>100	>100	>100	>100	0.601	0.492
NC7	>100	2.908	>100	>100	>100	0.497	0.450

**Table 5:** Emodel and MMGBSA energies of the binding of antifungal agents on cytochrome b based on MD simulations (based on two starting locations: L123_G137 and F129_G143).

L123_G137	MMGBSABinding Energy (kcal/mol)	Emodel_MD	F129_G143	MMGBSA Binding Energy (kcal/mol)	Emodel_MD
Ubiquinol	−112.872	−73.747	Ubiquinol	−126.815	−45.868
Fenamidone	−80.994	−43.187	Fenamidone	−76.720	−36.630
Famoxadone	−76.531	−59.682	Famoxadone	−54.023	−48.809
Mandestrobin	−61.791	−41.374	Mandestrobin	−60.710	−39.923
Azoxystrobin	−68.634	−57.910	Ametoctradin	−64.640	−38.293
Captan	−36.357	−25.133	Thiram	−52.433	−31.147
Thiram	−25.115	−32.646	Azoxytrobin	DNB	DNB
**L123F_G137**			**F129L_G143**		
Ubiquinol	−146.167	−78.895	Ubiquinol	−139.022	−53.130
Fenamidone	−74.073	−45.443	Fenamidone	−72.140	−43.909
Famoxadone	−92.628	−58.078	Famoxadone	−96.813	−48.809
Mandestrobin	−60.794	−38.257	Mandestrobin	−63.548	−36.662
Azoxystrobin	−60.789	−57.400	Ametoctradin	−36.603	−23.568
Captan	−51.978	−21.199	Folpet	−59.962	−32.186
Thiram	−35.643	−28.846	Thiram	−34.603	−37.411
			Azoxystrobin	DNB	DNB
**L123_G137A**			**F129_G143A**		
Ubiquinol	−137.872	−67.460	Ubiquinol	−92.641	−61.849
Fenamidone	−77.810	−45.726	Fenamidone	−56.688	−37.063
Famoxadone	−64.570	−57.756	Famoxadone	−62.046	−38.265
Mandestrobin	−63.381	−42.106	Mandestrobin	−52.780	−30.869
Azoxystrobin	−58.050	−55.299	Ametoctradin	−21.478	−26.039
Captan	−49.964	−28.915	Azoxystrobin	−41.085	−18.536
Thiram	−48.837	−24.120	Folpet	−47.835	−26.049
			Thiram	−51.838	−26.899
**L123F_G137A**			**F129L_G143A**		
Ubiquinol	−158.153	−73.468	Ubiquinol	−116.358	−57.339
Fenamidone	−60.840	−41.674	Fenamidone	−54.340	−42.041
Famoxadone	−68.740	−60.499	Famoxadone	−74.989	−46.843
Mandestrobin	−62.485	−42.106	Mandestrobin	−41.318	−41.520
Azoxystrobin	−43.013	−55.299	Ametoctradin	−35.546	−29.086
Captan	−48.054	−24.120	Thiram	−36.393	−32.139
Thiram	−42.518	−28.915	Azoxystrobin	DNB	DNB

## Data Availability

All data are included in the manuscript and supplementary data.

## Supplementary Materials

The following supporting information can be downloaded at: https://www.mdpi.com/article/10.3390/microorganisms11122966/s1.
