# Supplementary material for: Identification of Fungicide Combinations for Overcoming Plasmopara viticola and Botrytis cinerea Fungicide Resistance
Source: Microorganisms. 2023 Dec 12;11(12):2966. doi: 10.3390/microorganisms11122966 (PMC10746041; doi:10.3390/microorganisms11122966)
Supplement: Supplementary file 1 [file microorganisms-11-02966-s001.zip › microorganisms-2687386-supplementary.pdf]

Supplementary Data

Table S1. Compound Information for all the fungicides.

Compound #	Structure	Name	Molecular Formula
1	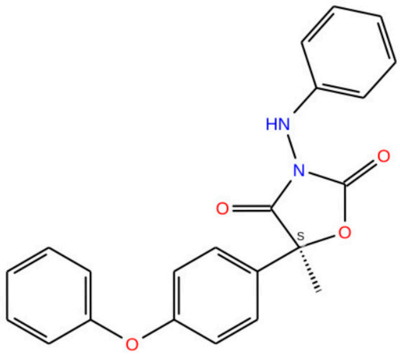	Famoxadone	$C_{22}H_{18}N_2O_4$
2	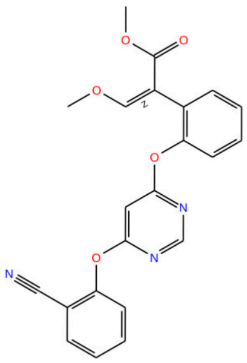	Azoxystrobin	$C_{22}H_{17}N_3O_5$
3	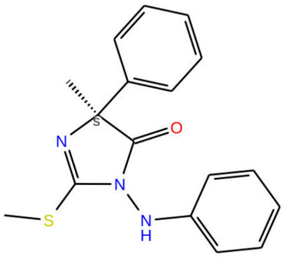	Fenamidone	$C_{17}H_{17}N_3OS$
4	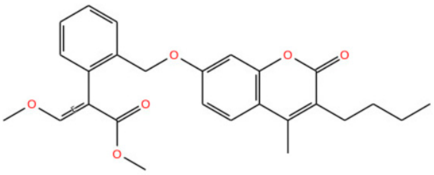	Coumoxystrobin	$C_{26}H_{28}O_6$

5	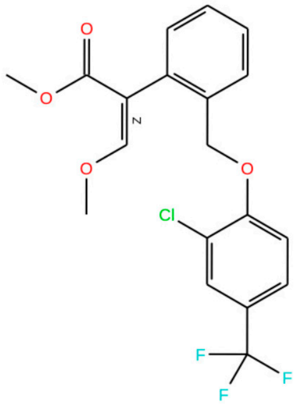	Flufenoxystrobin	$C_{19}H_{16}ClF_3O_4$
6	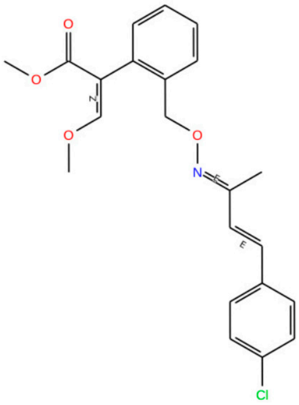	Enoxastrobin	$C_{22}H_{22}ClNO_4$
7	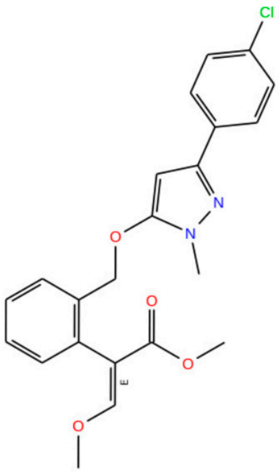	Pyraoxystrobin	$C_{22}H_{21}ClN_2O_4$

8	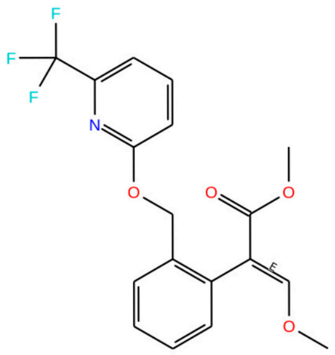	Picoxystrobin	$C_{18}H_{16}F_3NO_4$
9	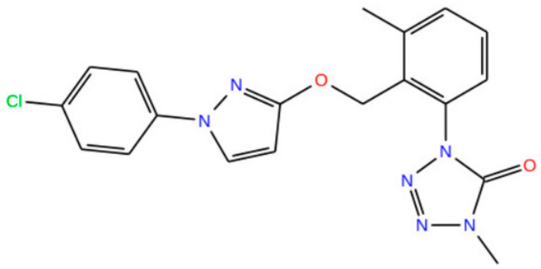	Metyltetraprole	$C_{19}H_{17}ClN_6O_2$
10	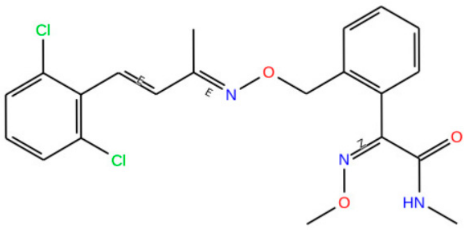	Fenaminstrobin	$C_{21}H_{20}Cl_2N_3O_3$
11	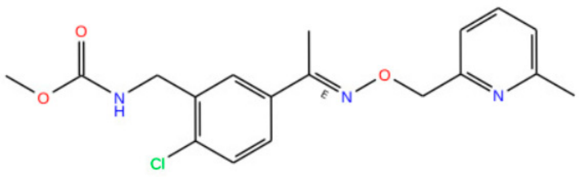	Pyribencarb	$C_{18}H_{20}ClN_3O_3$
12	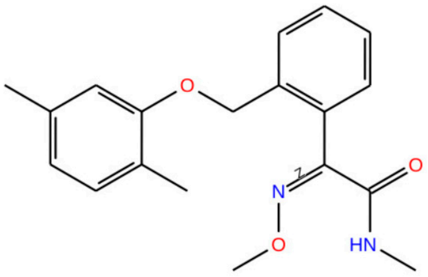	Dimoxystrobin	$C_{19}H_{22}N_2O_3$

13	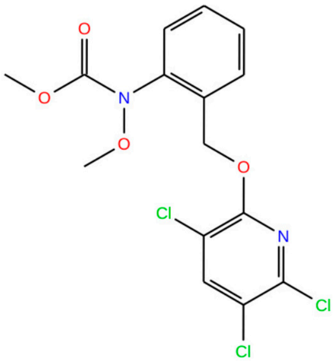	Triclopyricarb	$C_{15}H_{13}Cl_3N_2O_4$
14	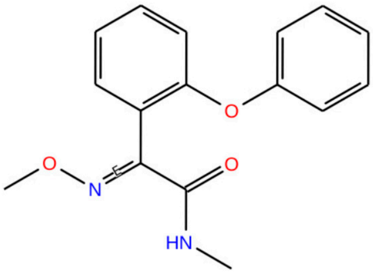	Metominostrobin	$C_{16}H_{16}N_2O_3$
15	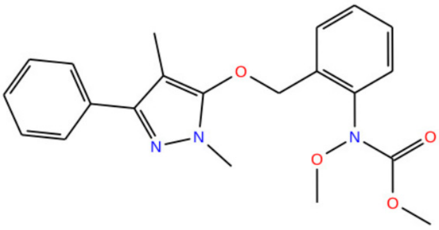	Pyrametostrobin	$C_{21}H_{23}N_3O_4$
16	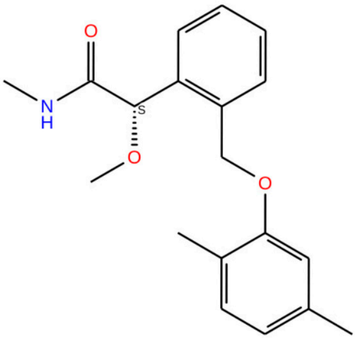	Mandestrobin	$C_{19}H_{23}NO_3$

17	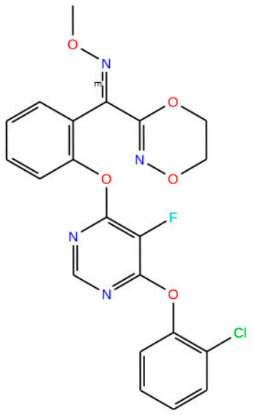	Fluoxastrobin	$C_{21}H_{16}ClFN_4O_5$
18	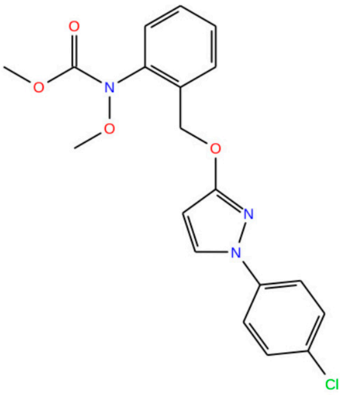	Pyraclostrobin	$C_{19}H_{18}ClN_3O_4$
19	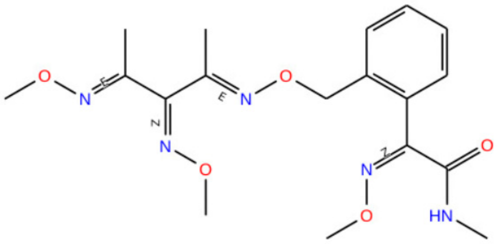	Orysastrobin	$C_{18}H_{25}N_5O_5$
20	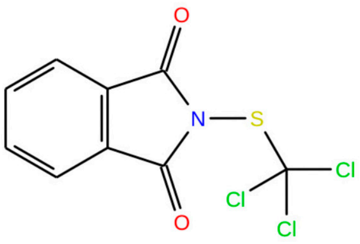	Folpet	$C_9H_4Cl_3NO_2S$

21	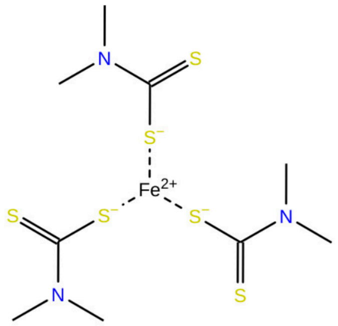	Ferbam	$C_9H_{18}FeN_3S_6$
22	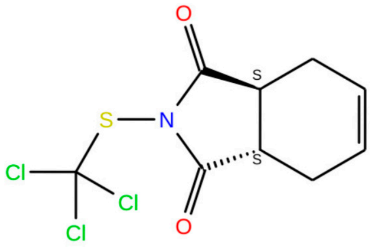	Captan	$C_9H_8Cl_3NO_2S$
23	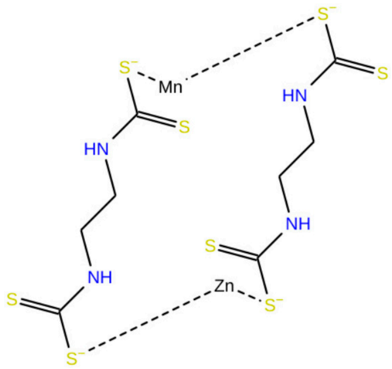	Mancozeb	$C_8H_{12}MnN_4S_8Zn$
24	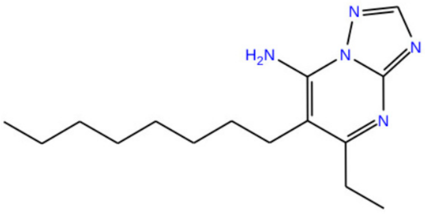	Ametoctradin	$C_{15}H_{25}N_5$
25	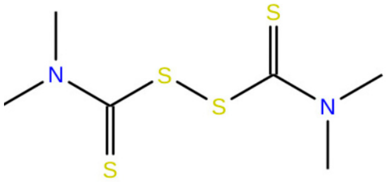	Thiram	$C_6H_{12}N_2S_4$
26	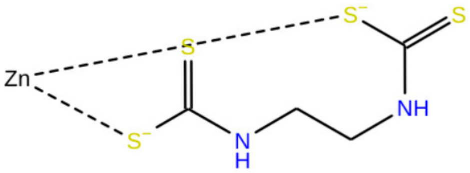	Zineb	$C_4H_6N_2S_4Zn$

A)

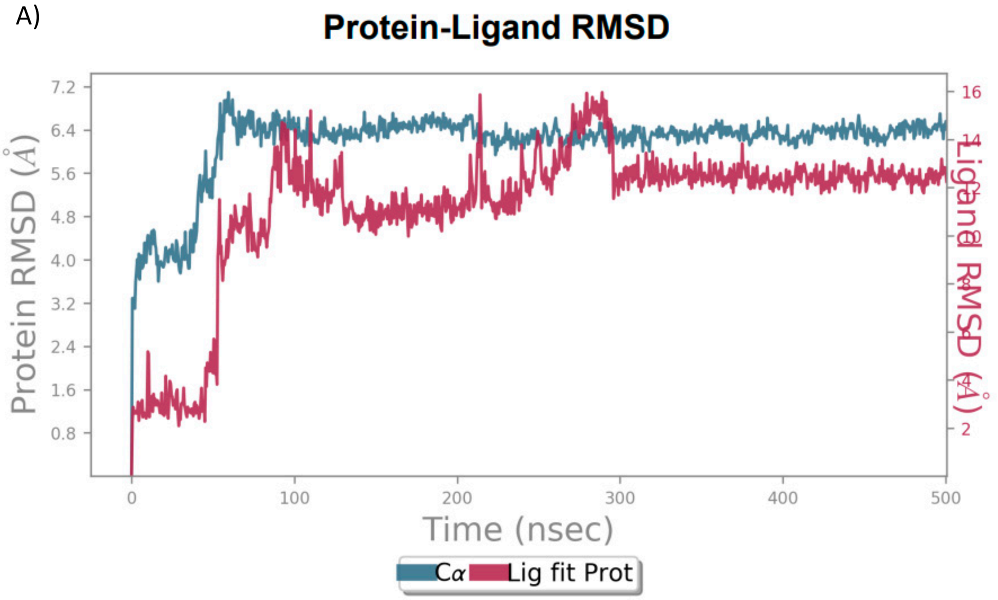

B)

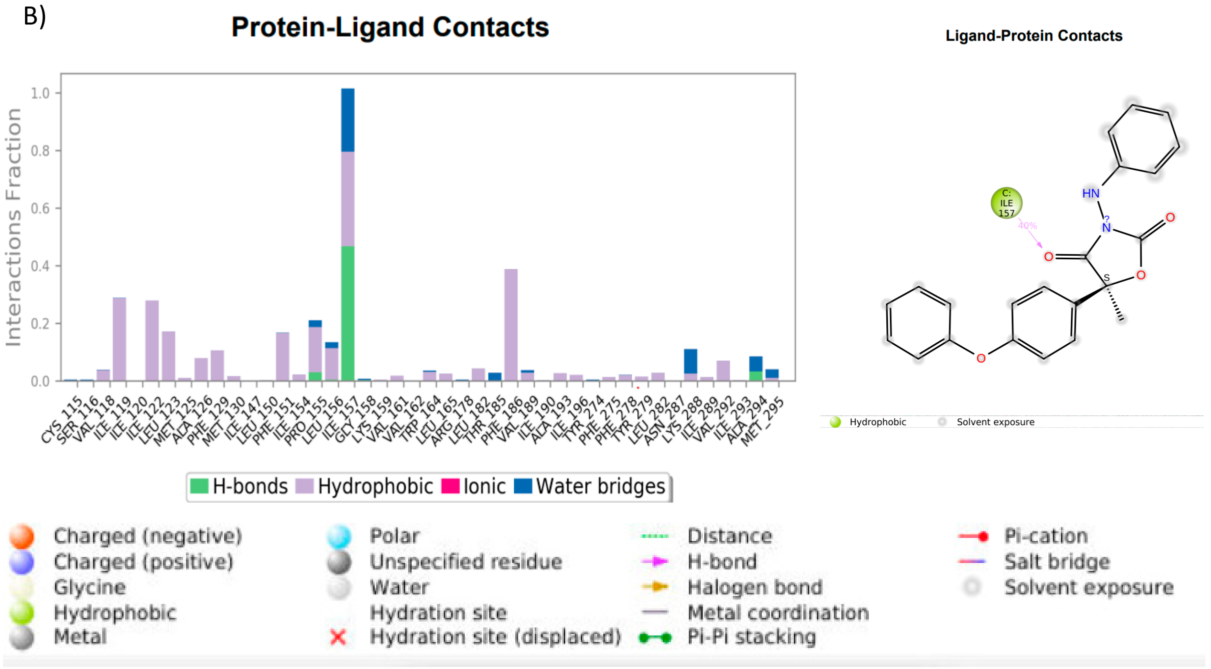

Figure S1. A) Protein and ligand RMSD for the trajectory of Famoxadone with WT cytochrome b of *Plasmopara viticola*. Protein RMSD is shown in dark blue and Ligand RMSD is shown in red. B) Protein-Ligand Interaction and contact of Famoxadone toward active sites F129 and G143 of *Plasmopara viticola* cytochrome b. X-axis represents active sites on cytochrome b; Y-axis represents simulation time of fungicide on specific active site.

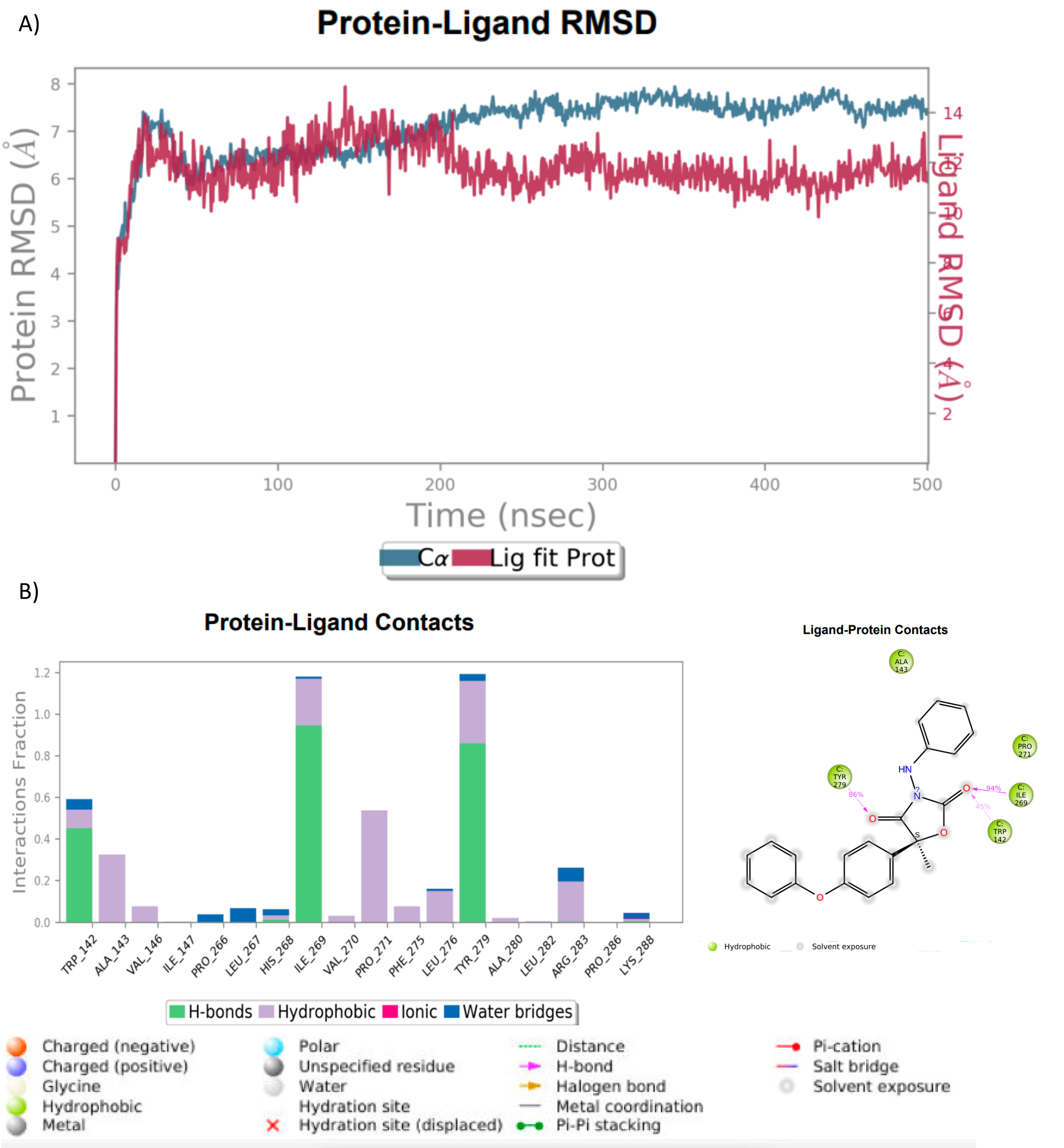

Figure S2. A) Protein and ligand RMSD for the trajectory of Famoxadone with G143A-mutated cytochrome b of *Plasmopara viticola*. Protein RMSD is shown in dark blue and Ligand RMSD is shown in red. B) Protein-Ligand Interaction and contact of Famoxadone toward active site G143A of *Plasmopara viticola* cytochrome b. X-axis represents active sites on cytochrome b; Y-axis represents simulation time of fungicide on specific active site.

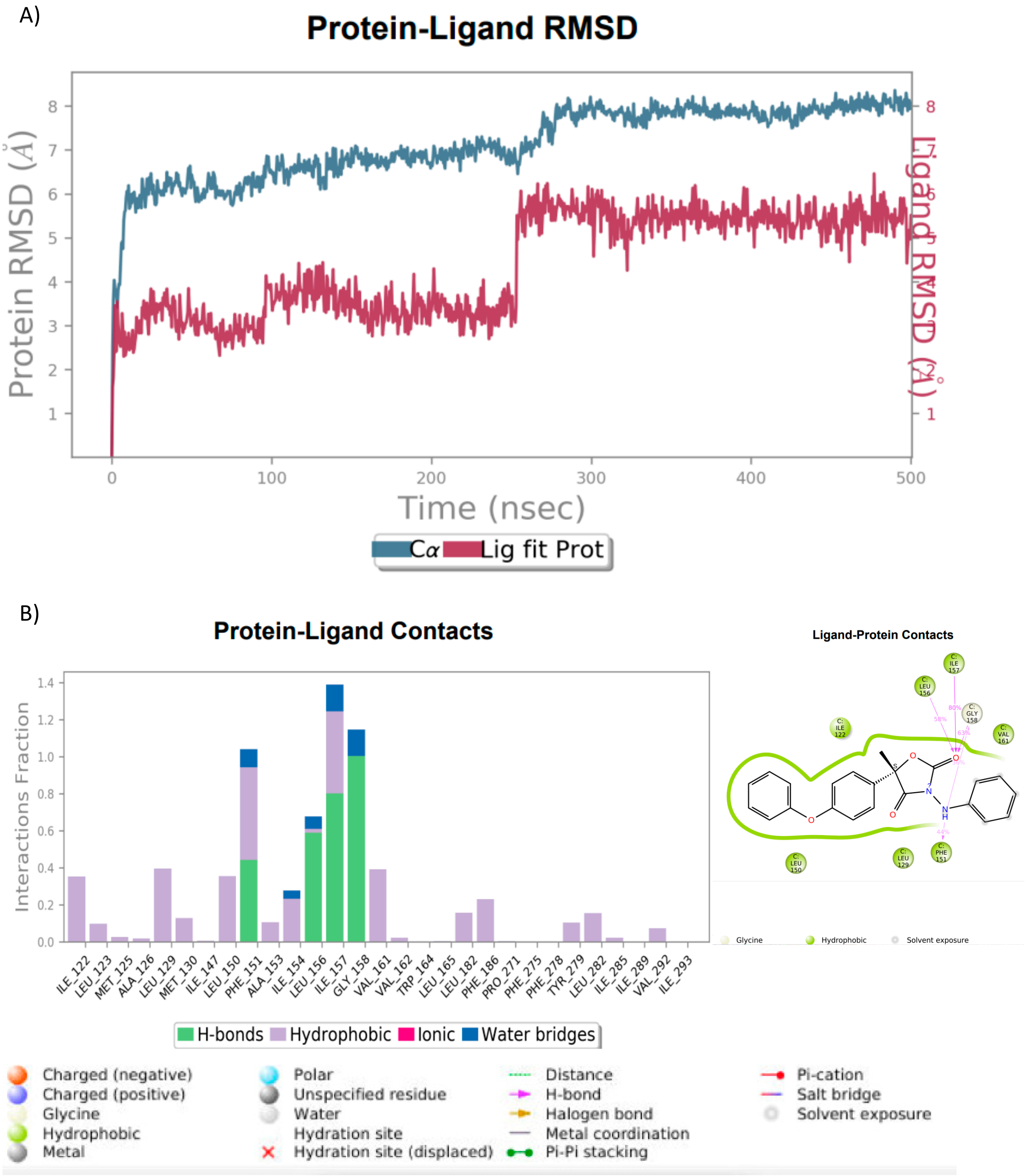

Figure S3. A) Protein and ligand RMSD for the trajectory of Famoxadone with F129L-mutated cytochrome b of *Plasmopara viticola*. Protein RMSD is shown in dark blue and Ligand RMSD is shown in red. B) Protein-Ligand Interaction and contact of Famoxadone toward active site F129L of *Plasmopara viticola* cytochrome b. X-axis represents active sites on cytochrome b; Y-axis represents simulation time of fungicide on specific active site.

A)

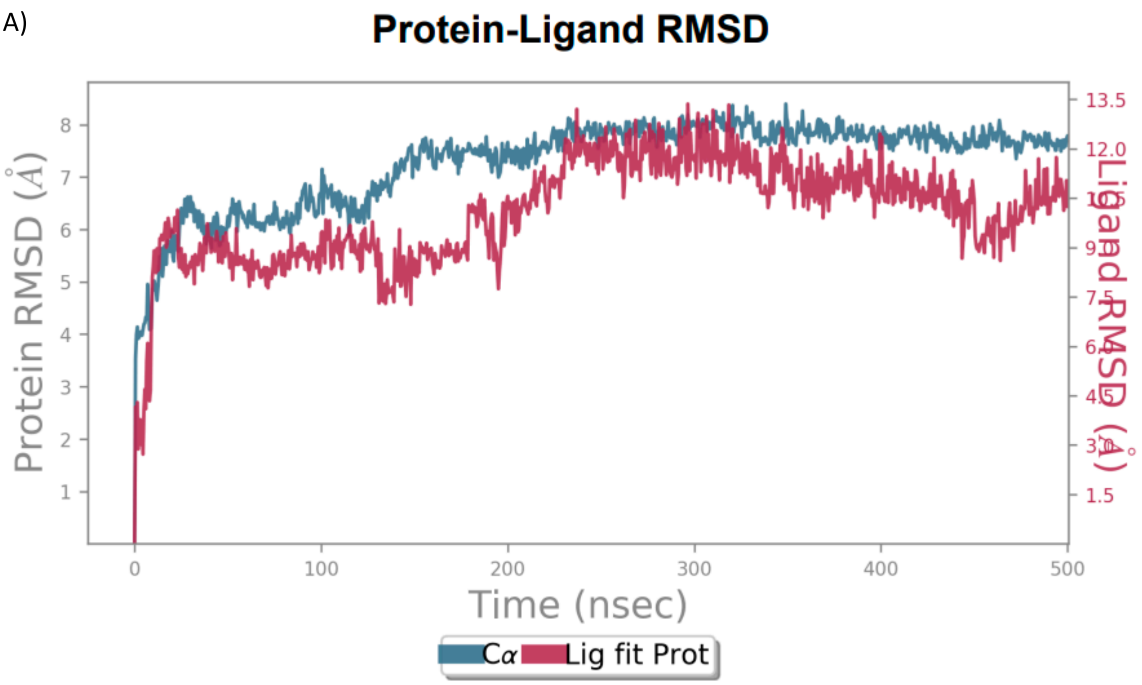

B)

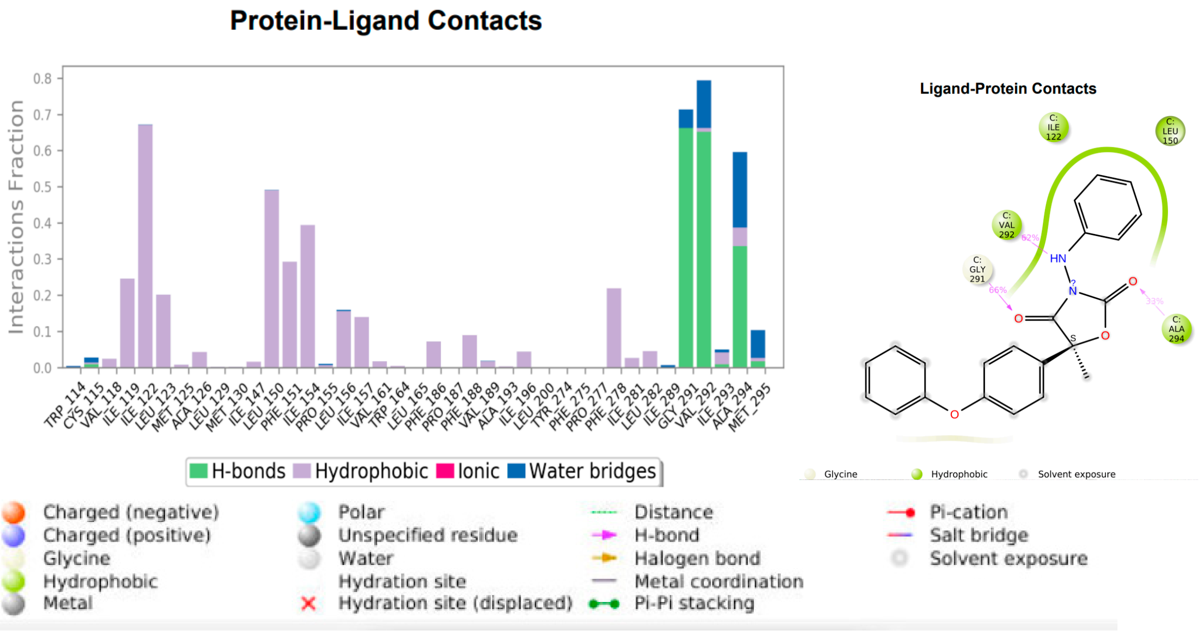

Figure S4. A) Protein and ligand RMSD for the trajectory of Famoxadone with G143A-F129L double mutated cytochrome b of *Plasmopara viticola*. Protein RMSD is shown in dark blue and Ligand RMSD is shown in red. B) Protein-Ligand Interaction and contact of Famoxadone toward active sites G143A and F129L of *Plasmopara viticola* cytochrome b. X-axis represents active sites on cytochrome b; Y-axis represents simulation time of fungicide on specific active site.

A)

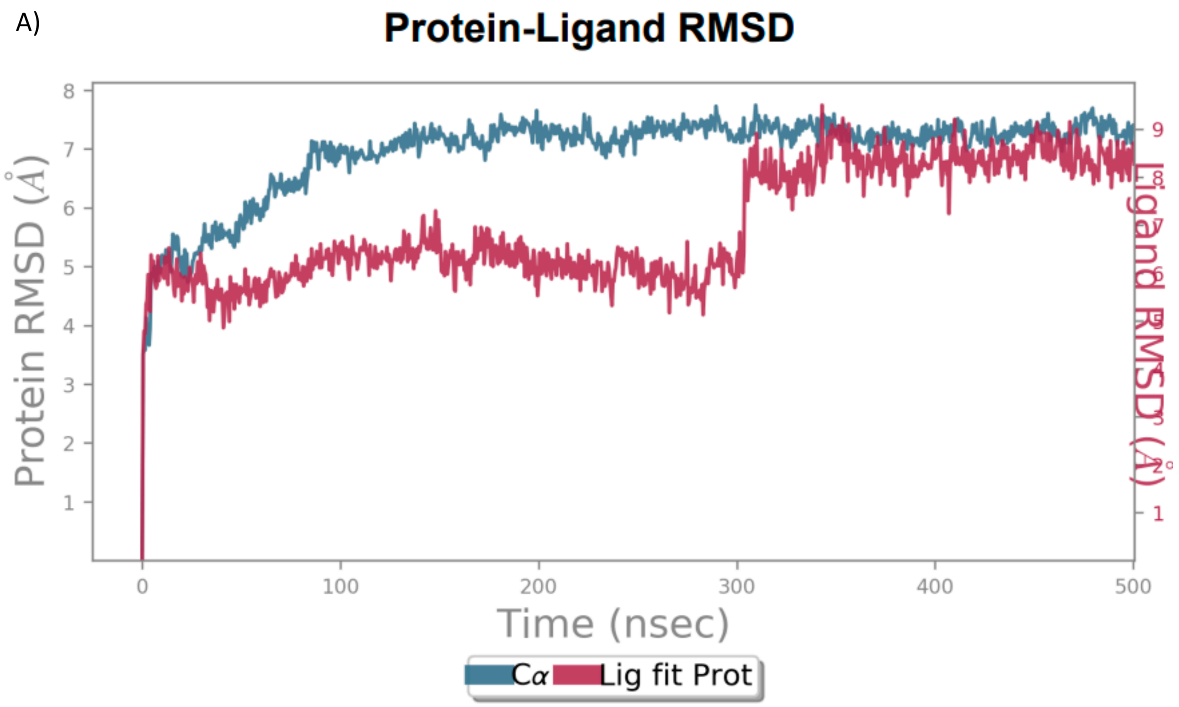

B)

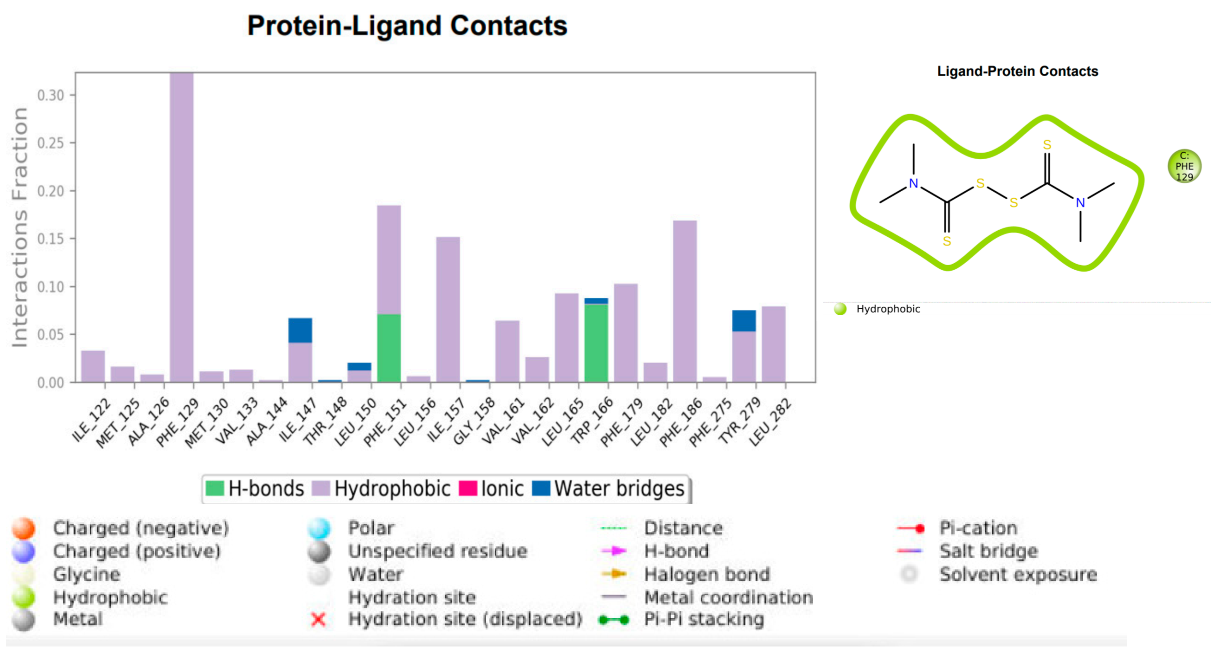

Figure S5. A) Protein and ligand RMSD for the trajectory of Thiram with WT cytochrome b of *Plasmopara viticola*. Protein RMSD is shown in dark blue and Ligand RMSD is shown in red. B) Protein-Ligand Interaction and contact of Thiram toward active sites G143 and F129 of *Plasmopara viticola* cytochrome b. X-axis represents active sites on cytochrome b; Y-axis represents simulation time of fungicide on specific active site.

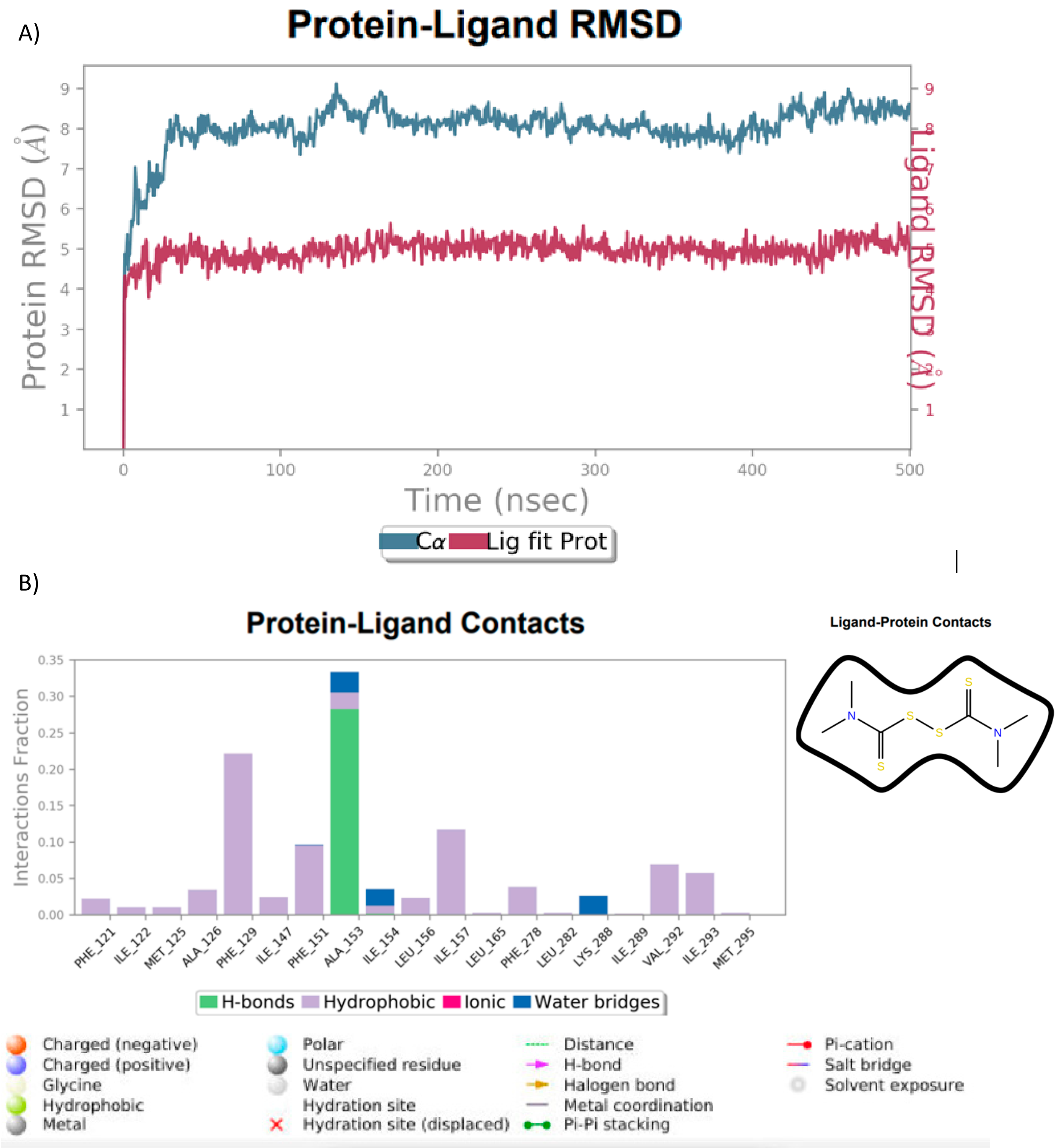

Figure S6. A) Protein and ligand RMSD for the trajectory of Thiram with G143A-mutated cytochrome b of *Plasmopara viticola*. Protein RMSD is shown in dark blue and Ligand RMSD is shown in red. B) Protein-Ligand Interaction and contact of Thiram toward active sites G143A of *Plasmopara viticola* cytochrome b. X-axis represents active sites on cytochrome b; Y-axis represents simulation time of fungicide on specific active site.

A)

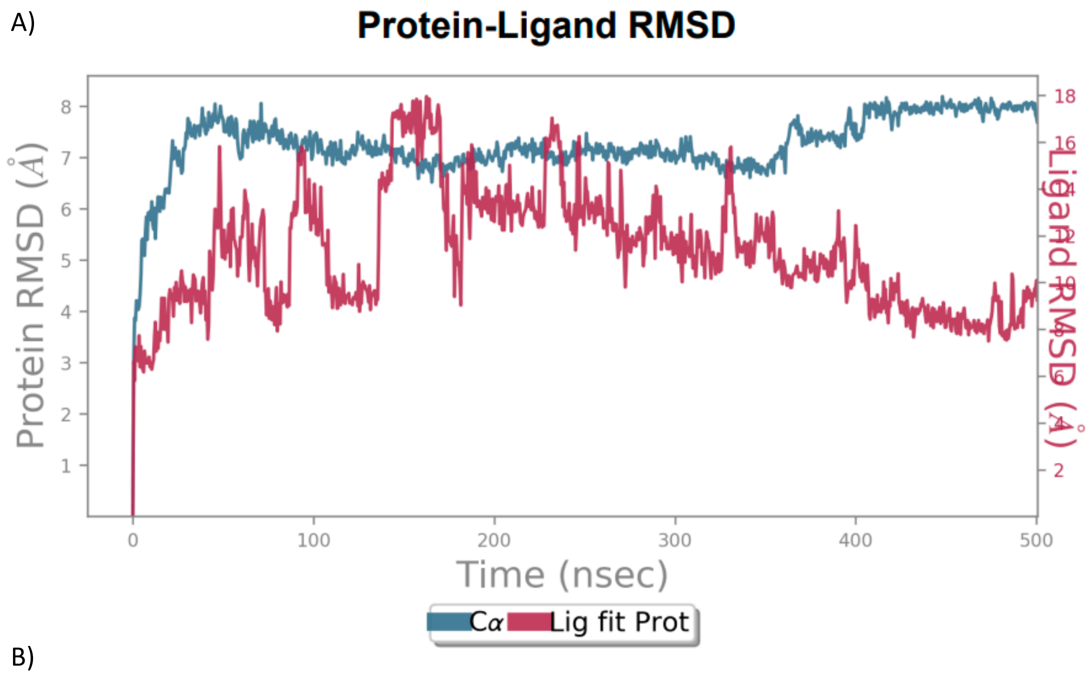

B)

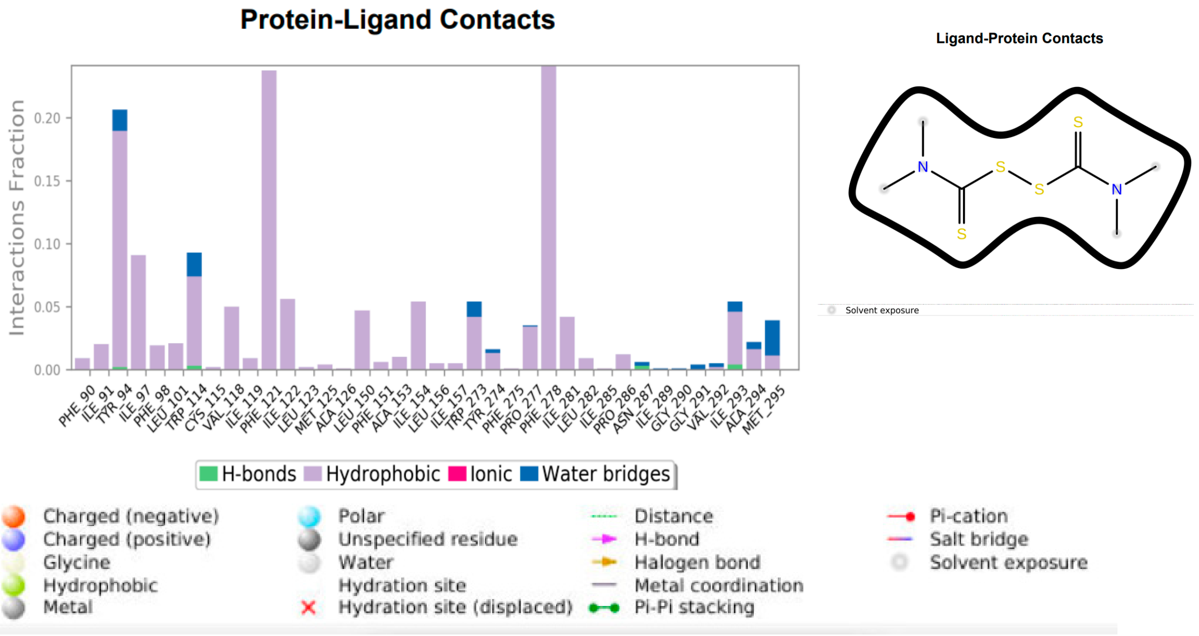

Figure S7. A) Protein and ligand RMSD for the trajectory of Thiram with F129L-mutated cytochrome b of *Plasmopara viticola*. Protein RMSD is shown in dark blue and Ligand RMSD is shown in red. B) Protein-Ligand Interaction and contact of Thiram toward active sites F129L of *Plasmopara viticola* cytochrome b. X-axis represents active sites on cytochrome b; Y-axis represents simulation time of fungicide on specific active site.

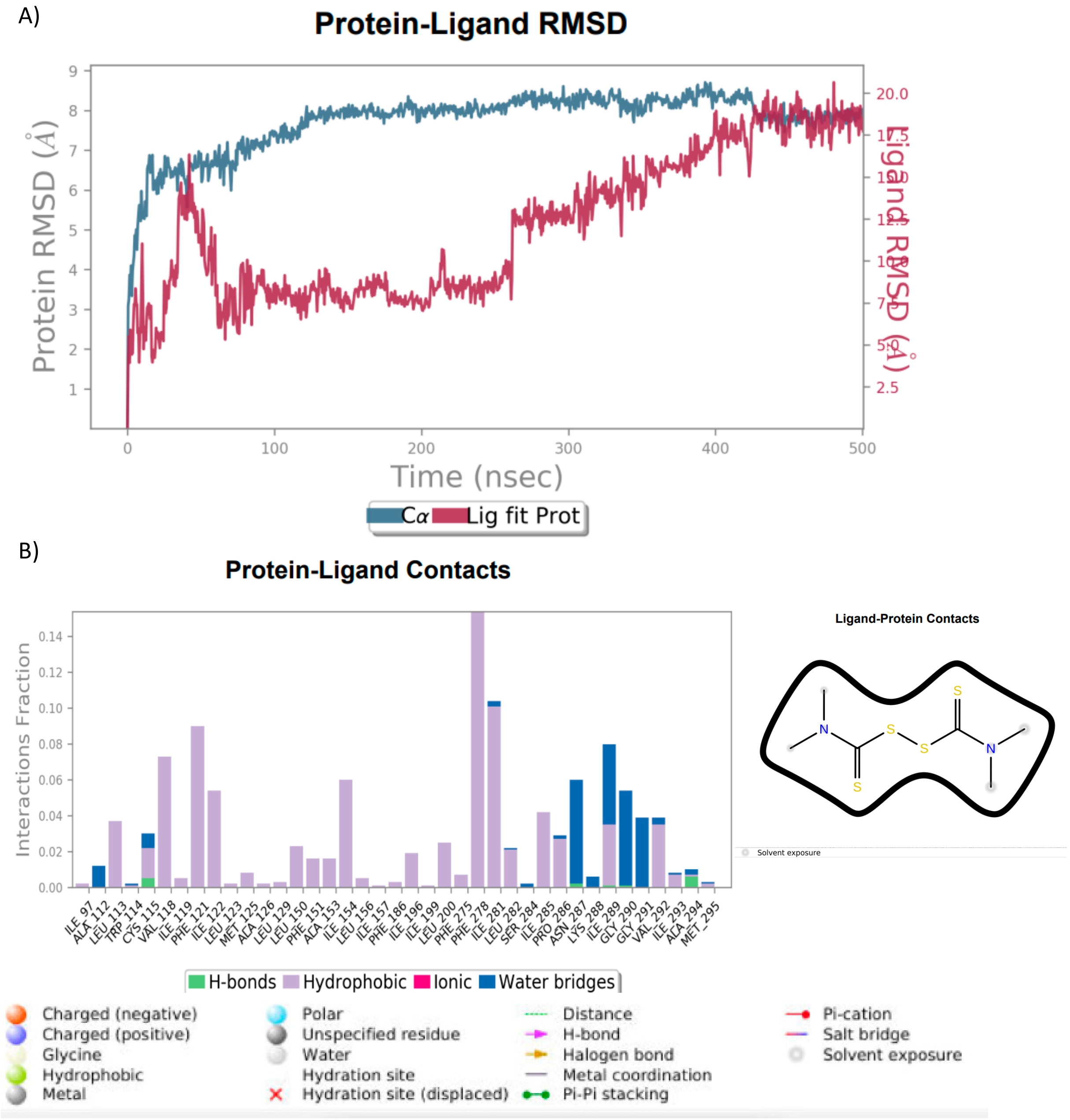

Figure S8. A) Protein and ligand RMSD for the trajectory of Thiram with G143A and F129L double mutated cytochrome b of *Plasmopara viticola*. Protein RMSD is shown in dark blue and Ligand RMSD is shown in red. B) Protein-Ligand Interaction and contact of Thiram toward active sites G143A and F129L of *Plasmopara viticola* cytochrome b. X-axis represents active sites on cytochrome b; Y-axis represents simulation time of fungicide on specific active site.

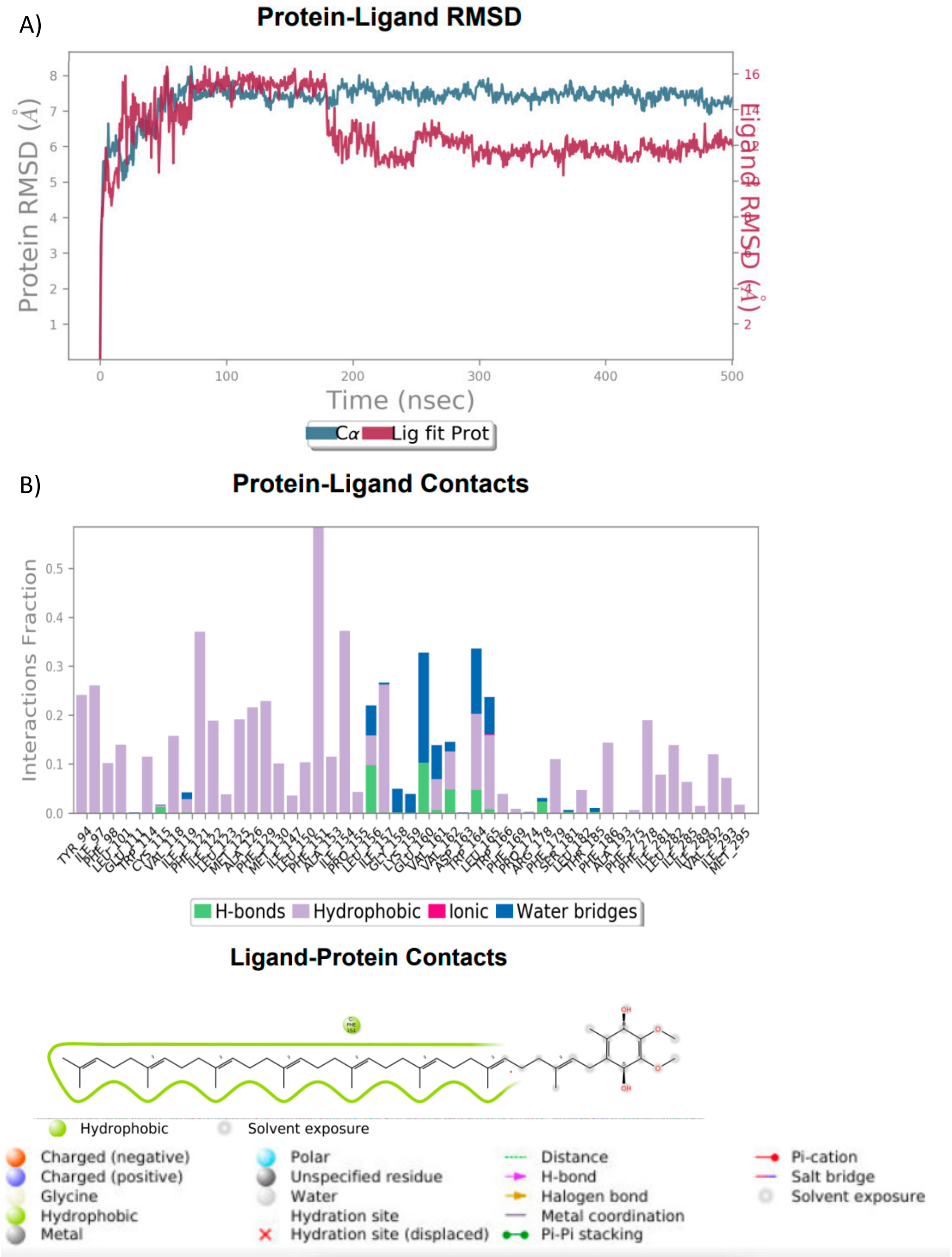

Figure S9. A) Protein and ligand RMSD for the trajectory of Ubiquinol with WT cytochrome b of *Plasmopara viticola*. Protein RMSD is shown in dark blue and Ligand RMSD is shown in red. B) Protein-Ligand Interaction and contact of Ubiquinol toward active sites G143 and F129 of *Plasmopara viticola* cytochrome b. X-axis represents active sites on cytochrome b; Y-axis represents simulation time of fungicide on specific active site.

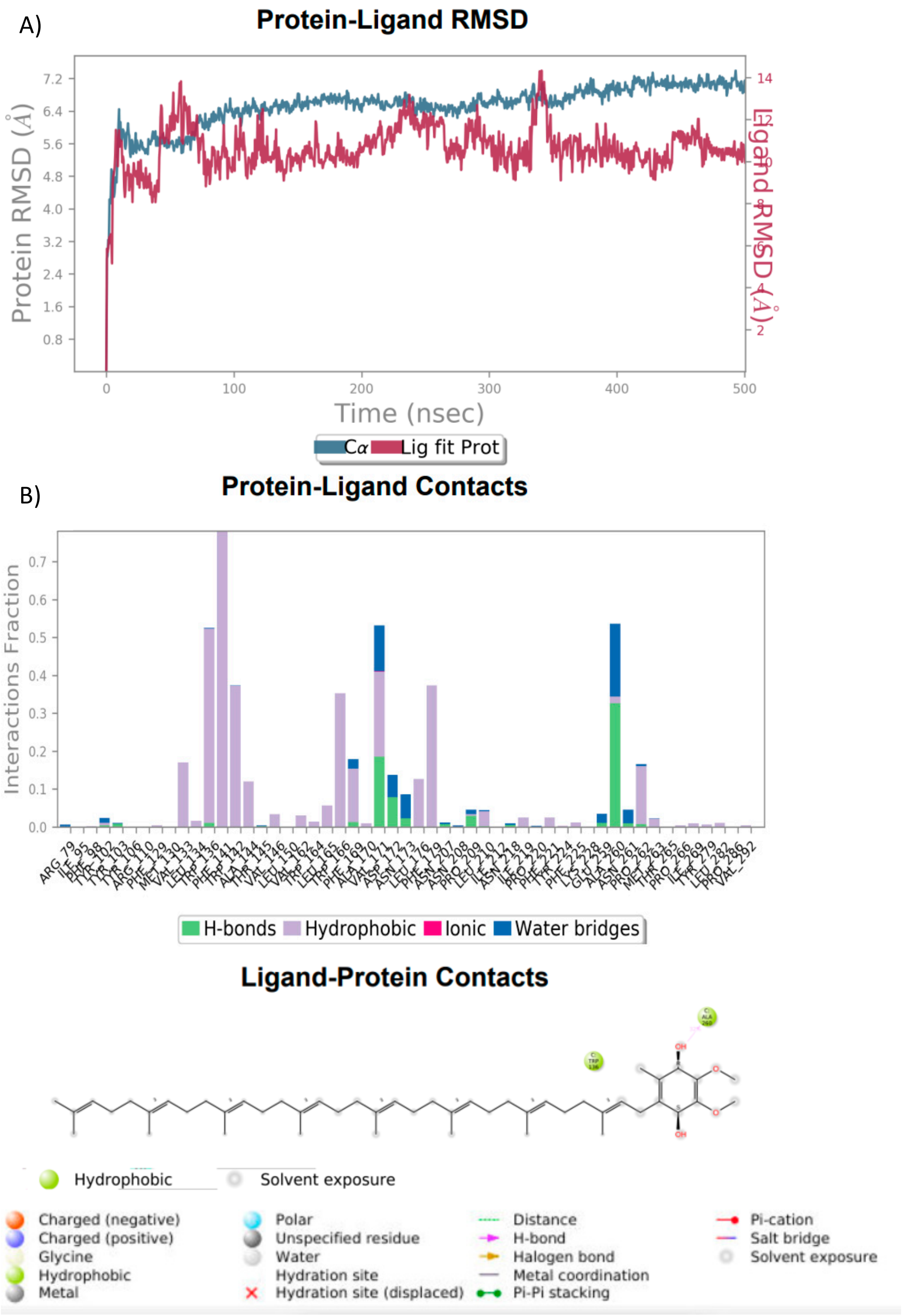

Figure S10. A) Protein and ligand RMSD for the trajectory of Ubiquinol with G143A-mutated cytochrome b of *Plasmopara viticola*. Protein RMSD is shown in dark blue and Ligand RMSD is shown in red. B) Protein-Ligand Interaction and contact of Ubiquinol toward active sites G143A of *Plasmopara viticola* cytochrome b. X-axis represents active sites on cytochrome b; Y-axis represents simulation time of fungicide on specific active site.

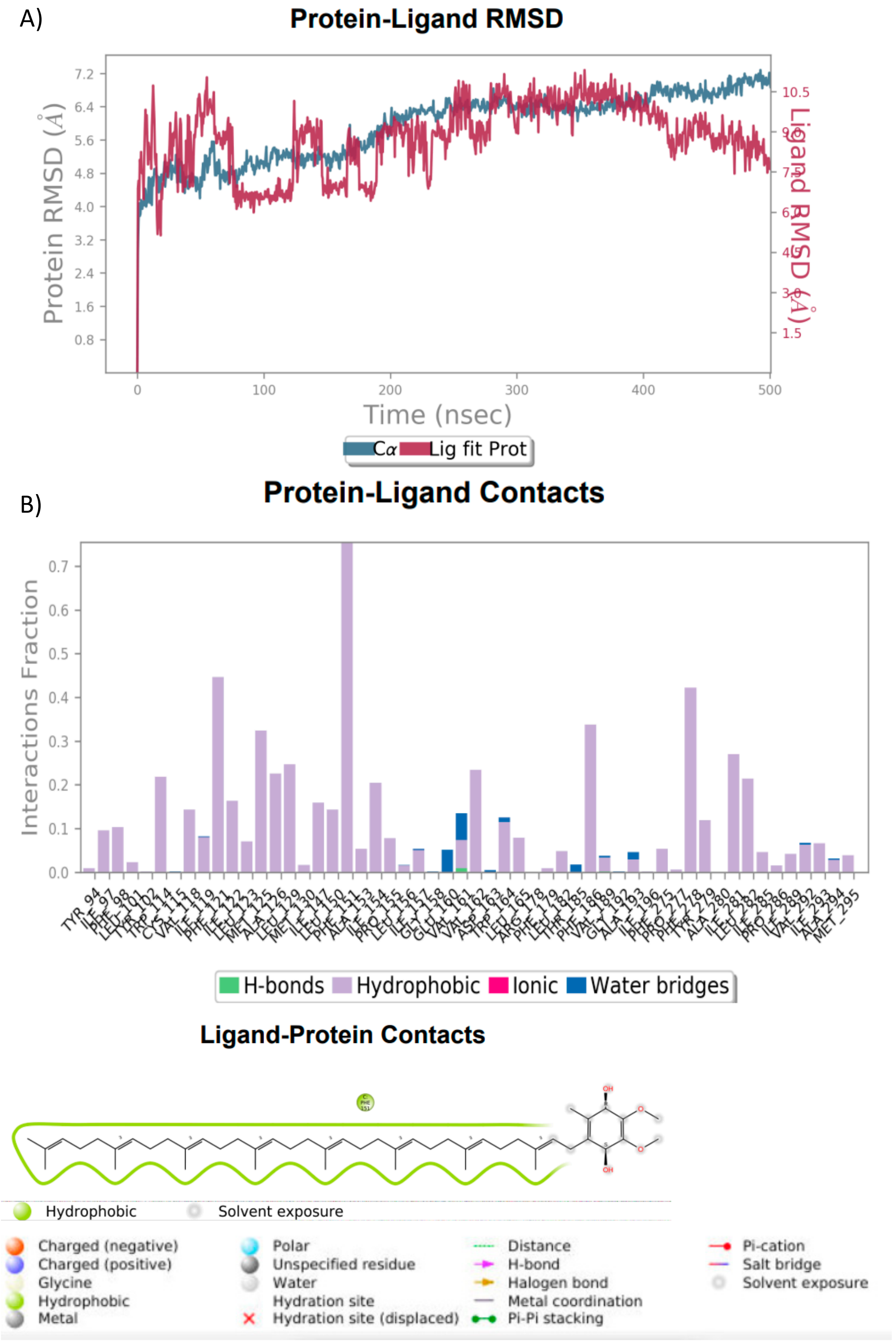

Figure S11. A) Protein and ligand RMSD for the trajectory of Ubiquinol with F129L-mutated cytochrome b of *Plasmopara viticola*. Protein RMSD is shown in dark blue and Ligand RMSD is shown in red. B) Protein-Ligand Interaction and contact of Ubiquinol toward active sites F129L of *Plasmopara viticola* cytochrome b. X-axis represents active sites on cytochrome b; Y-axis represents simulation time of fungicide on specific active site.

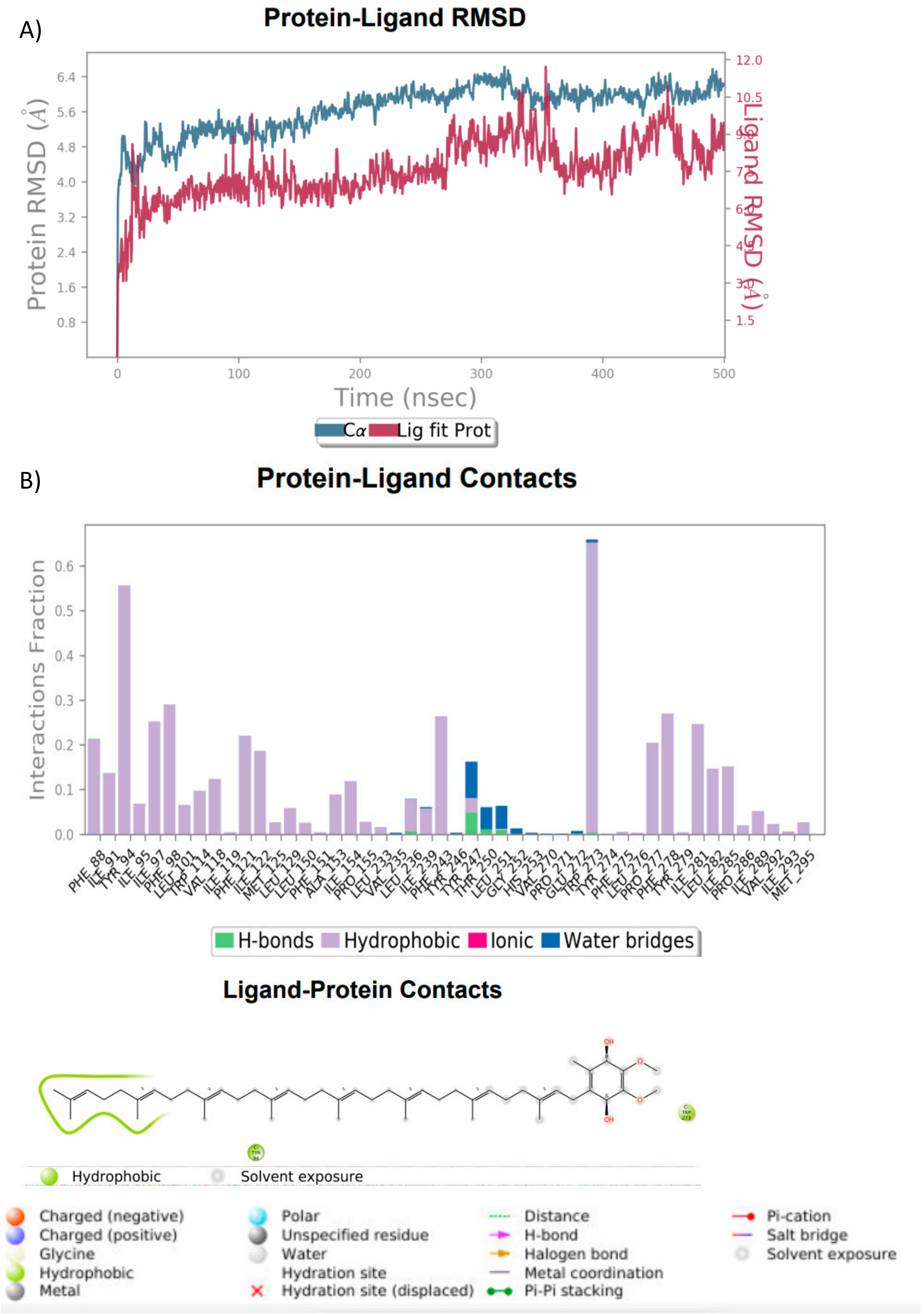

Figure S12. A) Protein and ligand RMSD for the trajectory of Ubiquinol with G143A-F129L double mutated cytochrome b of *Plasmopara viticola*. Protein RMSD is shown in dark blue and Ligand RMSD is shown in red. B) Protein-Ligand Interaction and contact of Ubiquinol toward active sites G143A and F129L of *Plasmopara viticola* cytochrome b. X-axis represents active sites on cytochrome b; Y-axis represents simulation time of fungicide on specific active site.
